# Machine learning approaches to optimize small-molecule inhibitors for RNA targeting

**DOI:** 10.1186/s13321-022-00583-x

**Published:** 2022-02-02

**Authors:** Hadar Grimberg, Vinay S. Tiwari, Benjamin Tam, Lihi Gur-Arie, Daniela Gingold, Lea Polachek, Barak Akabayov

**Affiliations:** Department of Chemistry and Data Science Research Center, Ben-Gurion University of the Negev, 8410501 Beer-Sheva, Israel

**Keywords:** Targeting RNA, Antibiotics, Small-molecule inhibitors, Chemical biology, Machine learning

## Abstract

In the era of data science, data-driven algorithms have emerged as powerful platforms that can consolidate bioisosteric rules for preferential modifications on small molecules with a common molecular scaffold. Here we present complementary data-driven algorithms to minimize the search in chemical space for phenylthiazole-containing molecules that bind the RNA hairpin within the ribosomal peptidyl transferase center (PTC) of *Mycobacterium tuberculosis*. Our results indicate visual, geometrical, and chemical features that enhance the binding to the targeted RNA. Functional validation was conducted after synthesizing 10 small molecules pinpointed computationally. Four of the 10 were found to be potent inhibitors that target hairpin 91 in the ribosomal PTC of *M. tuberculosis* and, as a result, stop translation.

**Graphical Abstract:**

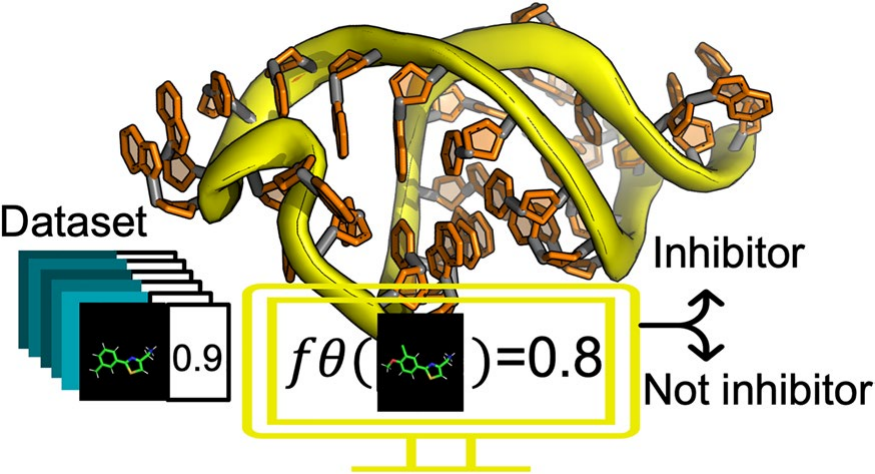

**Supplementary Information:**

The online version contains supplementary material available at 10.1186/s13321-022-00583-x.

## Introduction

A common practice in rational drug design involves molecular modeling in conjunction with optimization cycles taking into account structure–activity relationships (SAR). In such optimization cycles, certain functional chemical groups are replaced with others with the aim of improving the pharmacokinetics and potency of a drug candidate. This approach, known as bioisosteric replacement, has been common practice for more than 100 years, since Langmuir experimented with replacing atoms and functional chemical groups of certain molecules while retaining the molecules' physicochemical properties [1]. Indeed, the classic rationale underlying the design of active compounds with bioisosteres in the field of medicinal chemistry has not changed since that time. Nonetheless, the field has seen three major advances that have revolutionized not only molecular design and molecular recognition but also the synthesis of novel molecules, namely: (1) the availability of large virtual libraries of small molecules, (2) the development of computational algorithms that are able to exploit the big data resources of these virtual libraries, and (3) advances in computing power and in the utilization of graphic accelerators (GPUs), mainly on cloud servers, that facilitate the establishment and efficient examination of models built on the basis of the above virtual libraries and computational algorithms. Indeed, there is a growing body of literature that recognizes the importance of data-driven learning in drug discovery (a few examples of the large number of review articles on the subject are given in ref [2–8]).

**Fig. 1:**
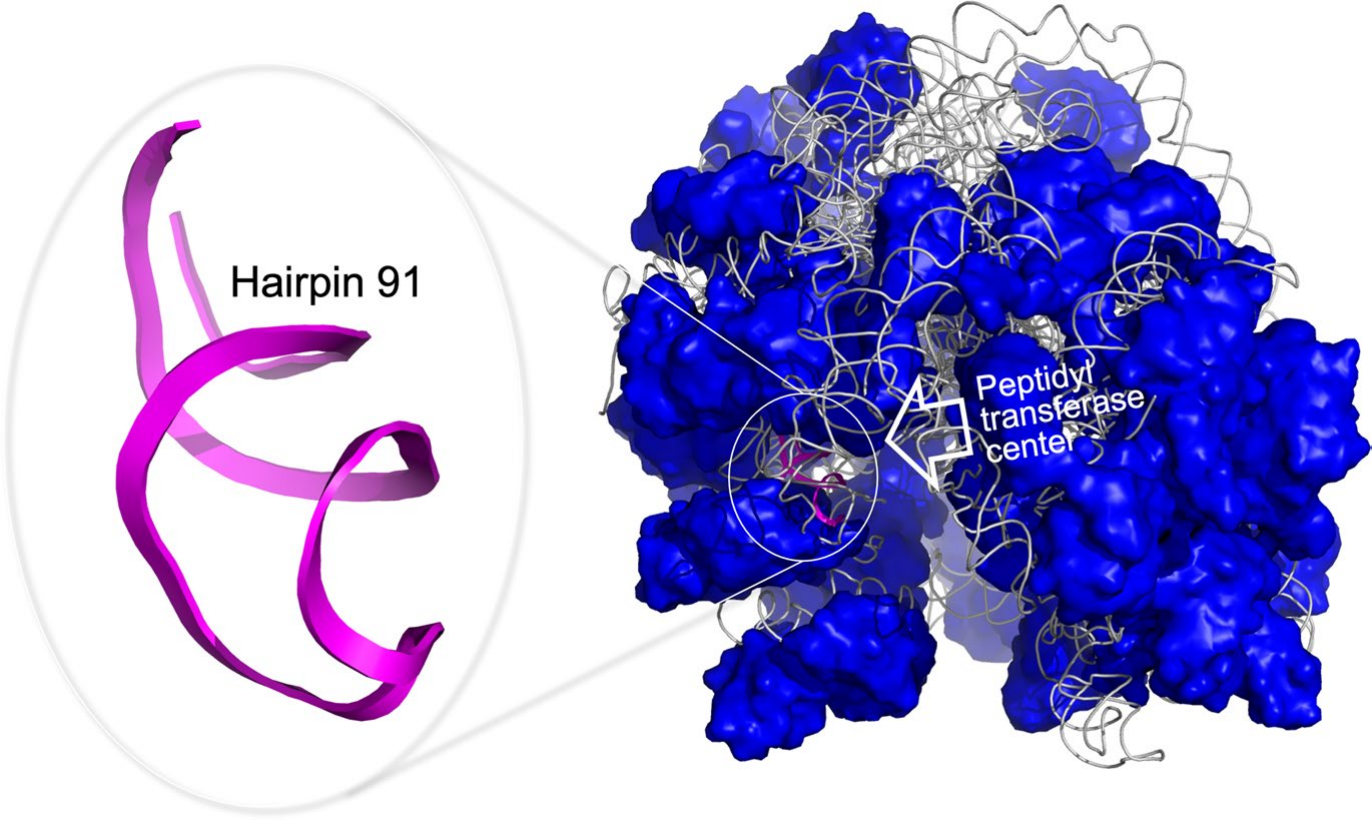
The complete structure of the *Mycobacterium smegmatis* 70S ribosome (PDBid: 5O61 [21]). Hairpin 91 used as a target for the design of small molecule inhibitors (purple) is part of the peptidyl transferase center. This hairpin is the RNA target that was used for the initial NMR fragment screening that yielded 2-phenylthiazole and for docking of 2-phenylthiazole containing drug-sized molecules

The starting point for the drug discovery study reported here is our previous work on the development of small-molecule inhibitors that target the ribosomal peptidyl transferase center (PTC) of *Mycobacterium tuberculosis*, the bacterium causing tuberculosis. The PTC of the *M. tuberculosis* ribosome is an important hub for protein synthesis and is hence an effective clinical target [9] (Fig. 1). The small-molecule inhibitors found in that study by applying a drug discovery pipeline that included NMR fragment-based screening all have 2-phenylthiazole as a common molecular scaffold. The straightforward binding of these molecules to a single PTC binding cleft yielded a reliable benchmark dataset containing the 3D molecular structures of nearly 800 small molecules with the 2-phenylthiazole moiety, together with their corresponding binding scores to hairpin 91 in the ribosomal PTC of *M. tuberculosis,* referred to below as the RNA target [9].

In the current study, we leveraged the three advances described above to develop an approach that combines different modern data-driven algorithms with the aim of extracting learning principles from our benchmark dataset to make predictions for better inhibitors of the RNA target and thereby to shorten the tedious optimization processes required for drug discovery. The design principles that we found were then used as guidelines for the synthesis of new inhibitors that target the ribosomal PTC of *M. tuberculosis*.

## Results and discussion

The main advantage of our benchmark dataset is that it relies on a simple target, hairpin RNA, that contains only one main binding cleft for small molecules and therefore limits the possibilities for binding (Fig. 1). Our dataset contains 791 drug-sized molecules with the 2-phenylthiazole moiety and their binding values for the RNA target. Although NMR screening to reveal the common scaffold has indeed decreased the space search for an effective molecular structure, it still remains challenging to rationalize molecular modifications by selecting specific features of the molecules that can boost target binding and, as a result, yield better inhibition. To address this issue, we therefore sought to extract learning principles by using data-driven algorithms, as is described below. Thereafter, candidate molecules were synthesized and subjected to biochemical validation.

**Fig. 2:**
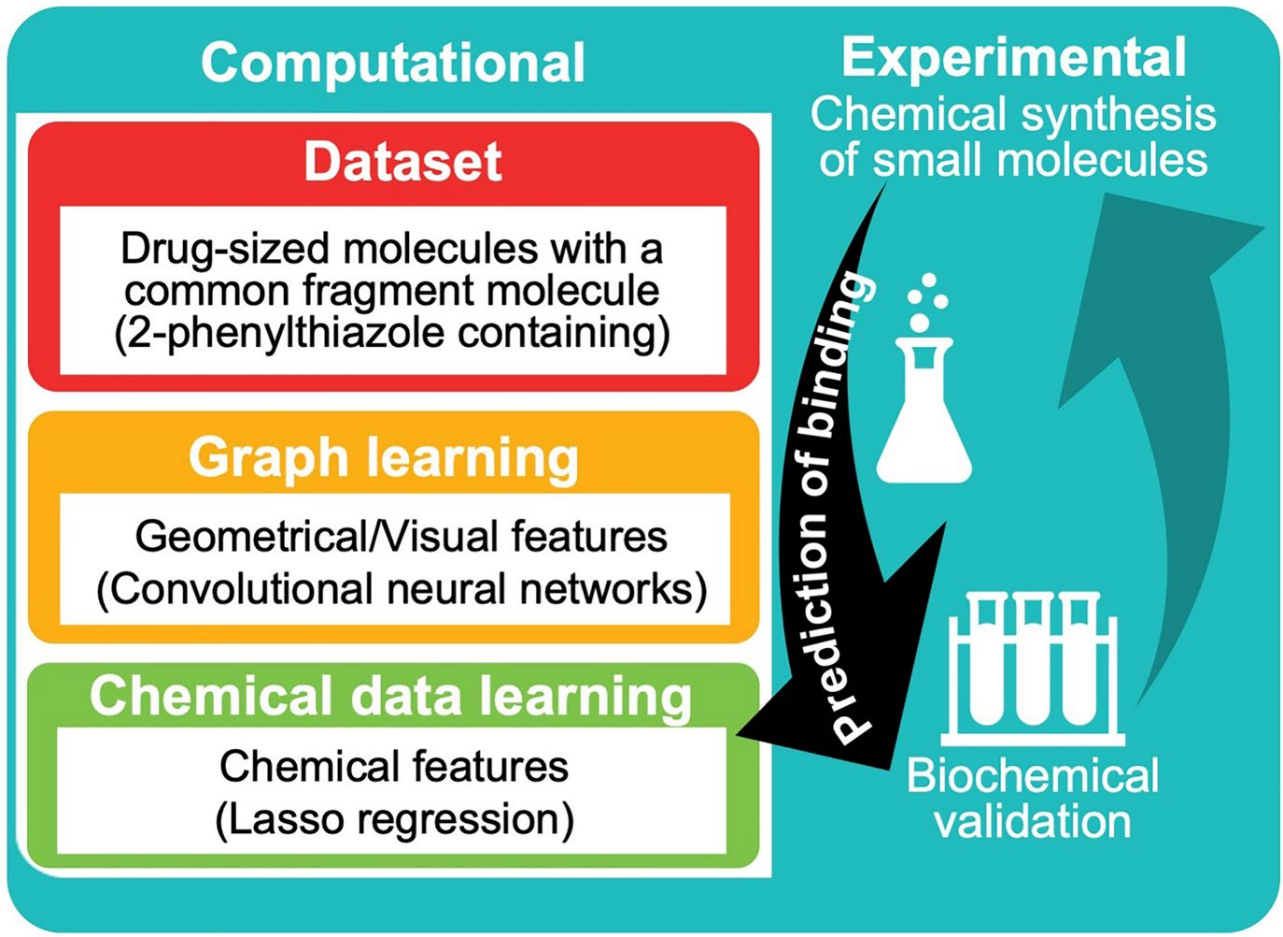
Scheme of the study. The study is divided into computational and experimental parts. In the computational part, deep convolutional networks, linear regressor, and classification trees were trained against a dataset of labeled small molecules. In the experimental part, small molecules with features important for RNA target binding were synthesized and then examined for their ability to inhibit ribosome activity (biochemical validation)

The study was thus divided into computational and experimental parts (Fig. 2). In the computational part, two principal categories of features were extracted from the molecules in the dataset: chemical features and geometrical/visual features. The latter are derived from sensory fields on the molecule that are stimulated upon activation by dominant coefficients of the model. Importantly, our data is labeled, i.e., every molecule in the dataset is assigned a binding value to the RNA target. Of relevance to this study, binding values were obtained [9] by virtual screening using the automated molecular docking program Autodock [10] on 791 filtered ZINC [11] molecules containing 2-phenylthiazole, the molecular scaffold that we previously identified by using transverse relaxation NMR spectroscopy [9]. In the current study we aimed to design better bioisosteric replacements for phenylthiazole-containing small molecules. To this end, we constructed different models to provide complementary insights into the principles that govern small-molecule binding to the RNA target. This data-driven design approach for discovering novel bioisosteric replacements facilitated the directed synthesis of molecules with the desired chemical modifications. For this purpose, we used three different machine-learning approaches, namely, the Lasso regression, decision tree classifier, and convolutional neural network (CNN) models, as described below.

### Machine learning models for the prediction of binding of small molecules to the RNA target

#### Lasso regression model

To determine the most suitable algorithm that most accurately predicts the binding scores of phenylthiazole-containing molecules with the aim of designing improved bioisoster replacements, we compared performances of various machine learning regressors (Additional file 1: Table S1). These algorithms were trained and estimated over our benchmark dataset that was divided into training and test sets (632 and 159 molecules, respectively). The algorithm that gave the best performance was linear regression with L1 regularization (Lasso); hence we sought to optimize this regressor. This algorithm allowed the crafting of chemical descriptors as functional fingerprints for the binding of small molecules to the RNA target. The most obvious advantage of exploiting chemical descriptors is that they are explanatory, meaning that one can reason that a particular inherent chemical property of a molecule would boost its activity. We extracted 204 chemical descriptors (features) from the small molecules in the dataset using the RDKit library [12]. Several methods were then used to sequentially reduce the number of features with the aim to improve the model’s performance. A final set of 32 features was selected as the optimal set of features (see *on-line Methods*, Feature engineering) that would produce the most accurate predictions.

**Fig. 3:**
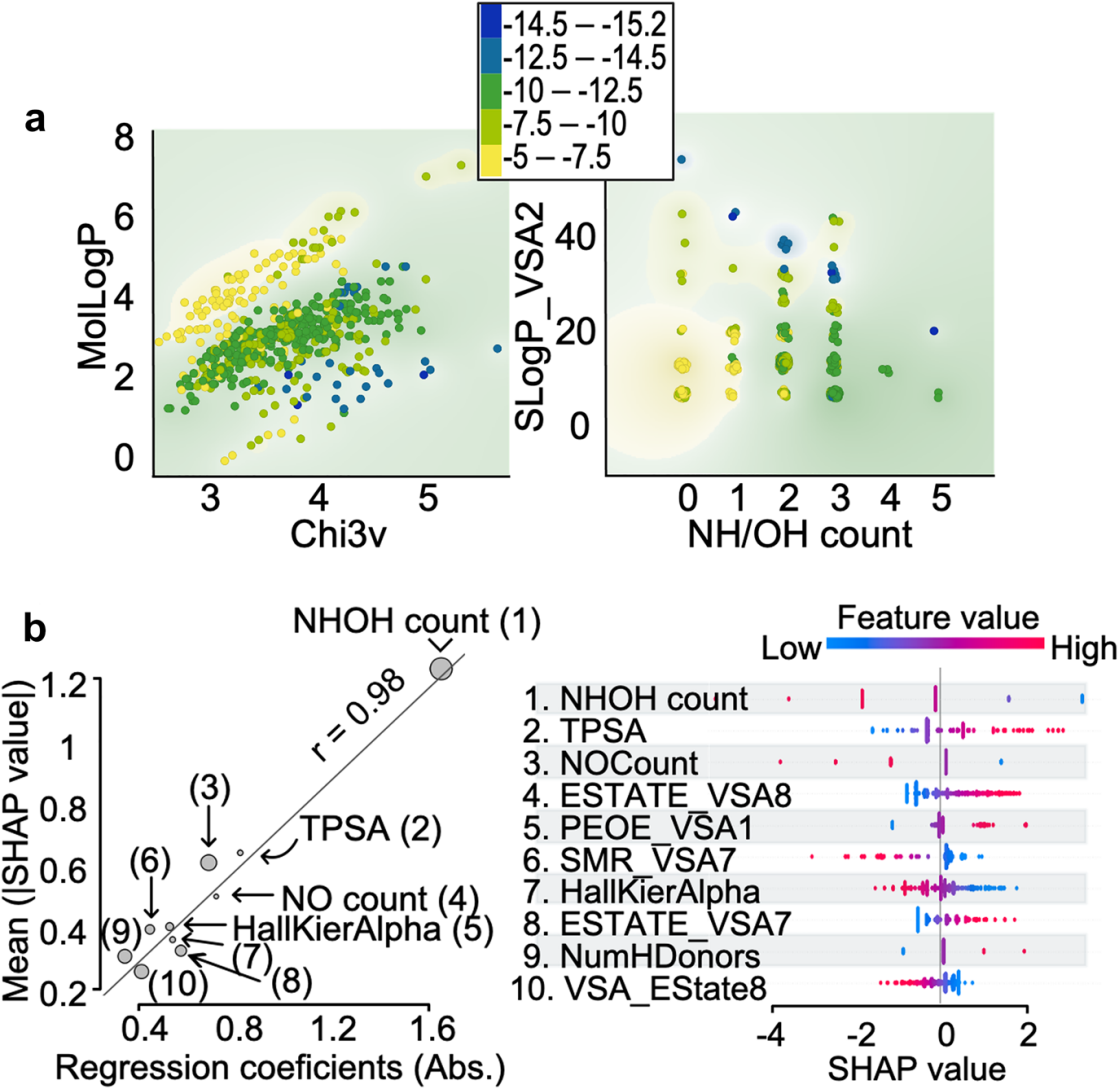
Importance of chemical features for the binding of small molecules to the RNA target. **a** Two features are sufficient to determine RNA binding: solubility (MolLogP) and regularity of the molecule (variance, Chi3v), respectively (left) or the hydroxy amino count (NHOH count) and subdivided van der Waal’s surface area (SlogP_VSA2 (right). The docking scores of the molecules range from − 5 (yellow) to − 15.2 (blue). The figure was created using an informative projection optimization function embedded in the Orange environment [22]. **b** Feature importance and inter-relationships between the top 10 features observed by the absolute regression coefficient (X-axis), SHapley Additive exPlanations value absolute mean values (SHAP [19], Y-axis), absolute correlation value of the feature with Y (point size). Feature importance observed by the distribution of SHAP values for every feature [14] is presented in the right panel. The 10 most influential features are: (1) NHOHCount—count of NH and OH groups in the molecule; (2) TPSA—topological polar surface area [23]; (3) NOCount—count of N and O atoms in the molecule; (4) Estate-VSA8—electrotopological state indices and van der Waals surface area [24] (2.05 ≤ x < 4.69); (5) PEOE_VSA1—capture direct electrostatic interactions based on atomic partial charge (− inf < x < − 0.30, y = 0) [25]; (6) SMR_VSA7—capture polarizability by molar refractivity with correct protonation state assumption (3.05 ≤ x < 3.63, y = 6) [25]; (7) HallKierAlpha—value for complexity, a topological descriptor for shape, size, and molecular complexity [15]; (8) Estate-VSA7—electrotopological state indices and van der Waals surface area [24] (1.81 ≤ x < 2.05); (9) NumHDonors—number of hydrogen bond donors; and (10) VSA_Estate8—topological state indices and van der Waals surface area [24] (6.45 ≤ x < 7.00). The Pearson correlation for all 32 features is presented in Additional file 1: Fig. S1

Examination of the relationships between the features revealed a clear pattern of molecular affinity, even for as small a number as two pairs of features (Fig. 3a, binding scores are represented by color code), indicating the importance of these features for the binding to the RNA target. The most prominent molecular features were found to be: (1) solubility (MolLogP), (2) regularity (variance of Ch3v) of the molecule, (3) hydroxy amino count (NHOH count), and (4) subdivided van der Waal’s surface area (SlogP_VSA2). These findings show that we can roughly classify the molecules in terms of their tendency to bind the RNA target by using a limited number of features.

After feature selection, we used a linear regression model to determine each feature's importance to the score prediction. The mean absolute error (MAE) loss for the test set was 0.424. The Pearson correlation (between y-true and y-predicted), computed to evaluate the model, was 0.951 (P-value = $7.22 \times 10^{ - 82}$). The importance of the molecular features was determined by the magnitude of the regression coefficients (for normalized data). After the training, we also obtained the SHapley Additive exPlanations value absolute mean values (SHAP) [13] (Fig. 3b), which measures the marginal contributions of each feature across all instances (molecules) to the prediction. In linear models, the SHAP value is the difference between the expected model output (mean of y true) and the partial dependence in the feature's value [14].

Since many features are highly interdependent, each feature's relative contribution to the model might deviate from the value obtained by correlation to the binding scores (size of points, Fig. 3b, left). The most important chemical feature for predicting the score that emerges from the two methods is the NH/OH count feature (Fig. 3b): The number of -NH and -OH groups in a given molecule determines the polarity or H-bonding for that molecule, which is presumably important for target binding.

#### Decision tree model

**Fig. 4:**
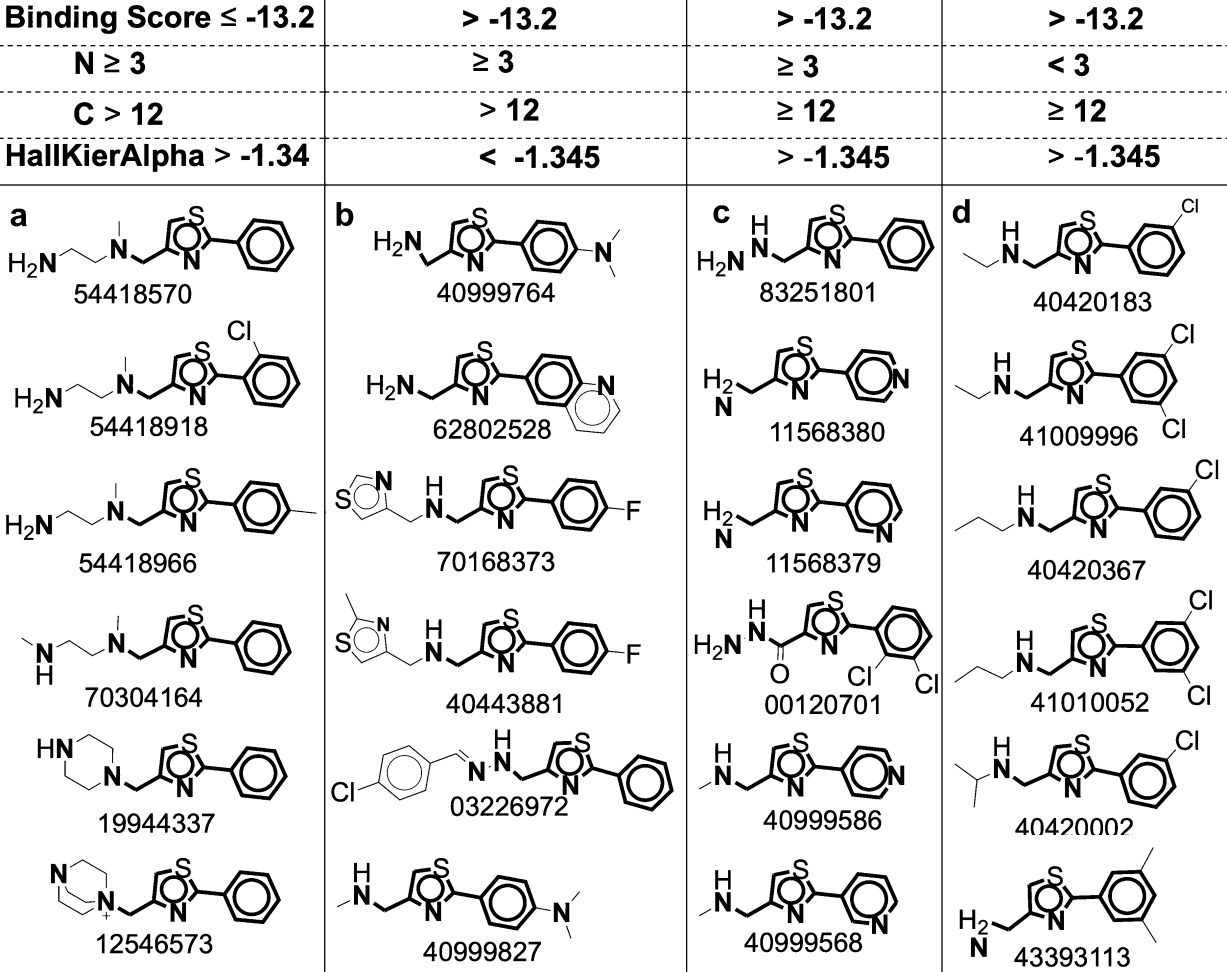
Groups of molecules based on the features revealed by the decision tree. The characteristics of each group are tabulated at the top of each column. Group a is the only group that has binding score of − 13.2 or better. It may be seen that the atomic bond that is connected to location 8 on the scaffold contains two nitrogen atoms and at least 3 carbon atoms, and no other kinds of atom (such as chlorine, fluorine, oxygen, etc.)

To better understand the molecular properties that govern the binding of small molecules to the RNA target, we simplified the prediction aim from predicting the binding score (regression) to predicting whether a molecule will bind or not (binary classification). We set a threshold above which the molecules in the top 2.5 percentile of docking scores (≤ − 13.2) were considered as binders. The decision tree classifier is highly explainable and relatively robust when using imbalanced datasets, and therefore we selected it as our model. Using feature selection algorithms (for additional details, see Methods), the number of features sufficient for predicting binding propensity of a molecule to the RNA target was reduced to as little as three: the number of nitrogen atoms (N) = 3, the number of carbons (C) > 12, and a value for complexity HallKierAlpha ≥ − 1.345 (where HallKierAlpha is a topological descriptor for shape, size, and molecular complexity [15]. Twenty-one of the 791 molecules fulfilled these criteria and were therefore considered as potential binders with binding score of − 13.2 or better (representative molecules belonging to this group are shown in Fig. 4, left column).

We previously found that location 8 in the phenylthiazole moiety is critical for binding to the RNA target [9]. The conclusions derived from the decision tree model provided additional insights into the most important molecular descriptors for binding of the RNA target. For example, a chemical determinant that includes two additional nitrogen atoms and at least three carbons, but no other atoms (such as chlorine, fluorine, or oxygen) can classify molecules with an excellent binding score to the RNA target.

#### CNN model

CNNs are neural networks that are designed to analyze visual data by pattern recognition. In this study, unique geometrical patterns in the small-molecule structure were analyzed with algorithms that are usually applied to identify patterns in larger-sized objects. Filters (2D or 3D) were evolved during model training to discover geometrical/visual features in the molecules. Specific activation maps formed by the convolutional layers were used for linking visual features in a molecule to its ability to bind to RNA. To examine the effect of input on the prediction, we used two types of molecular representation from our dataset: Simplified Molecular Input Line Entry System (SMILES, Additional file 1: Table S2) and low-resolution images (Additional file 1: Fig. S2).

#### CNN on SMILES representation

SMILES has previously been used as a digital format that describes the atomic arrangement, bonds, and other chemical properties of small molecules [16] (for the specific SMILES format that we used and the conversion to matrices, see Methods). The input of small molecules as SMILES strings in a CNN architecture has been used successfully to classify chemical activity and has revealed unknown, yet meaningful, chemical groups that affect the function of molecules [17]. Our model was trained with MAE loss to predict the binding score; the test loss achieved by the model was 0.484, and the test MAE was 0.479. The Pearson correlation (between y-true and y-predicted), computed to evaluate the model, was 0.934 (P-value = $4.63 \times 10^{ - 72}$). After training of the model, a receptive field, which refers to the part of the input that stimulates neurons along the network [18], was calculated for the entire dataset on the trained model (calculations were performed according to [19]). The substructure representing the calculated receptive field of the entire molecular matrix was extracted from the molecules (Additional file 1: Tables S3a, S3b).

**Fig. 5:**
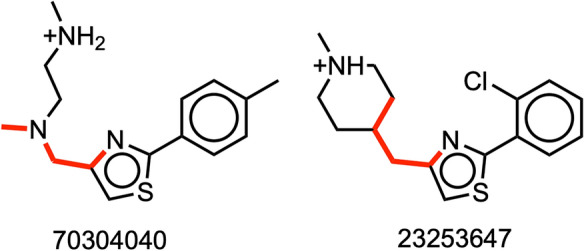
Visualization of the principal geometrical feature in molecules that bind to the RNA target. The substructure marked in red represents the most influential molecular motif of the binding score to the RNA target, as revealed by the CNN model

Filter 57 constitutes an influential sensing component in the network for inferring the substructure important for the RNA target binding. This filter yielded 18 molecules containing the same two significant substructures: N:C:1C([H])([H])N(C([H])); N:C:1C([H])([H])N1C([H]) (Fig. 5, Additional file 1: Table S4). If we consider 2.5% of the molecules (21 out of 791 molecules, two-sided confidence interval with 95% coverage) with the top binding scores as potential inhibitor molecules, then 17 out of these 18 molecules are part of this group (top 2.5%). Interestingly, the 17 molecules meet the criteria of the decision tree model. While filter 57 found substructures of molecules that contribute to the high binding score, filters 1, 9, 17, 22, 24, 28, 40, 52, 62 found substructures of molecules that contribute to the low binding score (Additional file 1: Tables S3a, S3b).

#### CNN on pictorial representations of molecular structures

The CNN model described above proved useful to extract features of the molecules where SMILES were used as input data representing the molecules. For purposes of comparison, we also used a CNN model with a different set of input visual representations of molecules comprising images. Our aim here was to develop a model that could be trained quickly without using intensive computing resources, i.e., we aimed to show that such a pictorial representation of the data, even with low resolution, is sufficient for accurate prediction of the binding scores of the molecule to the RNA target. The images therefore did not contain electrical charges, the molecule’s classification degrees of freedom, or the molecule’s conformation and polarity.

To examine the effect of different colors representing different types of atom in the molecular structure on the scores of binding to the RNA target, three types of image were compared: color, greyscale and binary (black/white) (Additional file 1: Fig. S3b). Colored images of the molecular structures provided only slightly better prediction [test MAE for RGB color 0.510, grayscale 0.556, and binary 0.580; Pearson correlation for RGB colors 0.881 (P-value = $7.59 \times 10^{ - 53}$), grayscale 0.839 (P-value = $2.01 \times 10^{ - 43}$), binary 0.839 (P-value = $2.77 \times 10^{ - 43}$)]. All the results were in the range of MAE of 5–6% of the docking scores and indicated no significant differences between the different image datasets (Additional file 1: Fig. S4).

Although the two types of input data, images and SMILES, gave similar overall prediction results, there were a few marked differences. The low-resolution of the pictorial input allowed significant acceleration of the model training and was computationally cost effective. However, SMILES provided chemical insights that can explain the binding of the molecules to the RNA target.

### Comparison between chemically driven models

We considered three different models: (1) a parametric model, in the form of linear regression, where feature importance was measured in terms of how its parameter vector interpreted as partial derivatives of the predicted variable with respect to the other features, (2) a non-parametric model, in the form of a decision tree, where feature importance was calculated by the probability of reaching a node compared to the probability of reaching other nodes within the same feature, and (3) CNN to reveal feature importance through a sequence of complex mathematical operations, both linear and nonlinear, that compute different aspects of the feature matrix through different kernels along the network. We thus sought to examine whether there was a connecting line between the results.

As mentioned above, the three most influential chemical features were revealed by the regression algorithm were: NH/OH count, TPSA, and N/O count. These features were examined within the substructures obtained by the filters. Except for the three substructures obtained by filter 9, no other substructures contained NH or OH, and therefore we could not explore this chemical feature across the models (means and SDs for the other features are presented in Additional file 1: Table S5). To compare the filters, a one-way ANOVA test was conducted for the two chemical features, TPSA and N/O count. The results revealed a main effect of filters on TPSA [F(9,194) = 47.24, p < 0.00001]. Post hoc t-tests with Bonferroni correction found that substructures from filter 57 had higher TPSA than each of the substructures from the non-inhibitory filters (Additional file 1: Fig. S5). Moreover, a main effect of filters on the N/O atom count was found [F(9,194) = 56.67, p < 0.00001]. Post hoc t-tests with Bonferroni correction found that filter 57 had a higher N/ O atom count than each of the non-binding filters (Additional file 1: Fig. S5).

### Design and synthesis of 10 phenylthiazole-4N-substituted piperazine hybrid molecules as novel inhibitors that target the ribosomal PTC of *M. tuberculosis*

We implemented the structural, pictorial, and chemical insights described above as design principles for the synthesis of new heterocycles with improved binding properties to the RNA target. Specifically, we designed molecules that contain 2-phenylthiazole connected at a piperazine nitrogen with either a carbonyl linker (**3b**, **4a–4c**) or a methylene linker (**6**, **7a–7e**) to alkyl, acyl or benzyl groups on the nitrogen of another ring (Additional file 1: Fig. S6, Table S7).

### Experimental corroboration of binding of newly synthesized molecules to the ribosomal PTC target

We used the molecular structure of the newly synthesized molecules as the test set for our machine learning models to predict binding to the RNA target and thereby inhibition of *M. smegmatis* ribosomes, where the non-pathogenic *M. smegmatis* was used as a proxy for the infectious *M. tuberculosis*. *M. smegmatis* ribosomes are often used to test antitubercular agents, such as those targeting the ribosomal PTC (U2673-C2836), since there is 100% identity of the PTC between *M. tuberculosis* and *M. smegmatis* (the identity of the whole 23S rRNA is 2873/3150, 91%). The regression and decision tree models were aimed at exploring the chemical features important for binding of the small molecules to the RNA target. The decision tree model also contributed to pinpointing the geometrical features of importance for binding to the RNA target. CNN on SMILES considered both chemical and geometrical features of the molecules, whereas CNN on images processed solely geometrical features. Importantly, visual and chemical features embedded in the molecules can be used for designing better small-molecule inhibitors. In addition, predictions using different trained algorithms must converge into the same overall conclusions and therefore raise the confidence in the results. We predicted the binding scores for the 10 newly synthesized molecules using the models trained and tested as mentioned above and compared these binding scores to the docking values (Autodock [10], Table 1). Out of these 10 newly synthesized molecules, four presented high docking scores, and only three were found to be good inhibitors in lab experiments, where a good inhibitor is considered as less than 7% ribosome activity. Ribosome activity was performed on *M. smegmatis* ribosomes in a bacterial coupled transcription/translation assay, in which the expression of luciferase gene was measured using luciferin assay (see Methods).

**Table 1:** Computational prediction and experimental verification of the 10 newly synthesized molecules

					Predicted binding score, model						
	R_1_	R_2_	X	Molecule (Zinc#)	Lasso^a^	CNN-SMILES^a^	CNN-pictorial^a^	Decision tree^b^	Docking values^a^	Ribosome activity (%)^c^	IC-50 (μM)
**1**	H	H	CO	32048999	− 8.53	− 7.92	− 8.18	II	− 10.75	100	No inhibition
**2**	**H**	**CH**_**3**_	**CH**_**2**_	**22780988**	**− 12.67**	**− 10.04**	**− 14.75**	**I**	**− 13.77**	**3.07**	**12.6**
**3**	H	COCH_3_	CO	44442665	− 9.93	− 7.97	− 8.18	II	− 7.49	100	No inhibition
**4**	**H**	**CH**_**2**_**CH**_**3**_	**CH**_**2**_	**19595411**	**− 14.05**	**− 13.81**	**− 14.96**	**I**	**− 13.73**	**1.19**	**9.1**
***5***	*H*	*Bn*	*CO*	*2992714*	*− 11.16*	*− 9.30*	*− 7.33*	*II*	*− 10.43*	*29.10*	*Weak inhibitor*
**6**	**H**	**Bn**	**CH**_**2**_	**22455064**	**− 13.91**	**− 13.90**	**− 9.89**	**II**	**− 15.29**	**2.21**	**9.77**
***7***	*H*	*Boc*	*CH*_*2*_	*914572967*	*− 10.55*	*− 9.83*	*− 12.96*	*II*	*− 10.26*	*69.87*	*Weak inhibitor*
***8***	*H*	*H*	*CH*_*2*_	*19944337*	*− 13.76*	*− 13.97*	*− 9.30*	*I*	*− 14.27*	*51.88*	*Weak inhibitor*
**9**	**H**	**COCH**_**3**_	**CH**_**2**_	**23218050**	**− 11.33**	**− 9.78**	**− 13.38**	**II**	**− 9.90**	**8.38**	**97.27**
**10**	Cl	Boc	CO	24030014	− 9.12	− 8.11	− 8.29	II	− 7.88	35.72	Weak inhibitor
Inhibitors recall					1.00	0.75	0.50	0.75			
Precision					1.00	1.00	0.50	1.00			
Accuracy					1.00	0.90	0.70	0.90			

^a^Negative score corresponds to stronger binding; bold—effective inhibitor, italics—weak inhibitor

^b^Group classification: binding (group I) > binding (group II), groups are based on decision tree classifier (Fig. 3)

^c^Activity measured in the presence of 1 mM of the compound and *M. smegmatis* ribosomes in a bacterial coupled transcription/translation assay

For the decision tree and CNN on SMILES models, three molecules out of the four were predicted to be binders (Table 1), whereas regression predicted that all four molecules would bind strongly to the RNA target. CNN on images, however, identified only two molecules as inhibitors. CNN on SMILES revealed that among the 10 newly synthesized molecules seven contained the substructures revealed by filter 57. Out of the seven molecules revealed by filter 57, three were predicted as strong binders and three were predicted as weak binders to the RNA target. Note that experimental results of ribosome activity was yet another crucial step in validating the models trained with labels obtained from docking.

The inhibitory effect of the 10 synthesized compounds (**1–10**, Table 1) on *M. smegmatis* ribosomes was tested in a bacterial coupled transcription-translation assay in which the expression of the luciferase gene was determined (see Methods [20]). The results indicated effective inhibition of *M. smegmatis* ribosomes by four molecules (Table 1).

## Conclusion

This study set out to design new small molecule inhibitors by applying state-of-the-art computational optimization models. We used complementary models that combine deep CNNs with classical machine learning regression and decision tree models. To complement and validate the computational work, the predicted molecules were synthesized and tested for their ability to inhibit translation by targeting the RNA hairpin in the ribosome. Our findings showed that both chemical and geometrical considerations can be taken into account in the optimization. Regression analysis using chemical features extracted by RDkit revealed that binding of our drug-sized small molecules to the RNA target is influenced largely by higher N/NH and O/OH counts whereas lower TPSA values are more common in inhibitors’ substructures than in non-inhibitors’ substructures. Higher count of N/NH and O/OH may be related to the elevated potential of H-bonding as well as other features related to the van der Waals surface area and the electrostatic potential, were found to be important for the establishment of better molecule-RNA binding. The influential features derived from the decision tree model are N ≥ 3 atoms, > 12 C atoms, and HallKierAlpha > − 1.345, descriptors that are related to shape, size and complexity of the molecules which contribute to better molecule-RNA target binding. In addition, comparative analysis revealed that CNN emerges as a reliable predictor for small molecule binding by using input data of either images or 3D structures. Although CNN is considered less explanatory, a receptive field yields the most important substructures processed by each filter in the network. CNN on images revealed that robust and accurate binding scores can be obtained almost simultaneously for multiple molecules and using limited computational resources. The results obtained with low-resolution images indicate that very little information is needed to provide accurate predictions and that the differences between using color, grayscale, or binary images are not significant.

These findings contribute in several ways to our understanding of targeting RNA with small molecules and provide a basis for smart design of new small molecule inhibitors, as follows: (1) the use of a limited but robust dataset is sufficient to reach reliable chemical optimization, (2) a reduction in the degrees of freedom for binding is crucial in the case of the hairpin RNA, which has only one binding cleft, and (3) SARs obtained computationally must be validated experimentally.

Application of the models to molecules synthesized in the laboratory yielded similar predictions in most cases—the models had accuracy rate of 70–100%. In general, our findings have significant implications for the understanding of how small-molecule inhibitors bind to their targeted macromolecule. Although this study focuses on RNA in the context of specific antibiotic agents, the findings may well have a bearing on targeting other types of RNA or other macromolecules for the development of therapeutics against a large range of human diseases.

## Methods

### Materials

All chemicals and reagents were purchased from Sigma Aldrich Chemical Company (St. Louis, MO) and Acros Organics (Fair Lawn, NJ), and were used without further purification or drying. The progress of the reactions was monitored by thin-layer chromatography (TLC) on ready-made silica gel plates (Merck, Darmstadt, Germany; UV active). The plates were either developed under iodine vapor or visualized directly under UV-light (254 nm). Column chromatography was performed over flash silica gel (60A size). ^1^H-NMR spectra were recorded at 400 MHz or 500 MHz and ^13^C-NMR spectra were recorded at 100 MHz or 125 MHz using Bruker (Billerica, MA) DRX-400 and Bruker DRX-500 spectrophotometers, respectively, and reported in parts per million (ppm) on the δ scale relative to tetramethylsilane as the internal standard. Coupling constants (J) are reported in Hz.

### Design and synthesis of ten phenylthiazole-4N-substituted piperazine hybrid molecules

Benzothiamides were reacted with 2-bromo-ethyl pyruvate to form the corresponding 2-phenylthiazole 4-ethyl ester derivatives (**2a** and **2b**). The esters were hydrolysed and reacted with N-Boc-piperazine using DCC and HOBt to give corresponding amides (**3a** and **3b**). The N-boc deportection of amide (**3a**) followed by reaction with acetic anhydride or benzyl bromide, provided carbonyl linked phenylthiazole-4N-substituted piperazine hybrid molecules (**4a**–**4c**). On the other hand, the ester (**2a**) was reduced using sodium borohydride to alcohol **5** and converted into the corresponding mesylate with methansulfonyl chloride and triethylamine. The mesylate was then substituted with N-Boc piperazine to give intermediate compound **6**. Finally, N-boc-deprotection and reaction with acetic anhydride or benzyl bromide followed by reduction using lithium aluminium hydride provided methylene linked phenylthiazole-4N-substituted hybride molecules (**7a–7e**).

### General procedure for synthesis of phenyl thiazoles 2a and 2b

Ethyl bromopyruvate (1.8 ml, 12 mmol, 1.2 equiv) was added to a stirred solution of thiaobenzamide derivatives **1a** or **1b** (10 mmol) in ethanol (30 ml) at room temperature. The reaction mixture was heated under reflux for 5 h, and the progress of the reaction was monitored by TLC. The solvent was removed by evaporation at reduced pressure, and the residue was washed with water and extracted with ethyl acetate (3 × 30 ml). The combined organic layer was dried over anhydrous sodium sulfate, filtered, and evaporated. The residue was purified by silica gel column chromatography using ethyl acetate hexane (1:9) as the eluent to afford the desired product **2a** and **2b** (70–90%) as a white solid.

### Synthesis of compounds 3a and 3b

To a stirred solution of compound **2a** or **2b** (3 mmol) in THF (10 ml) and ethanol (15 ml) was added an aqueous solution of lithium hydroxide (1.2 M, 5 ml). After stirring at room temperature for 4–5 h, the volatiles were removed under reduced pressure. The residue was acidified with 1 N hydrochloric acid to pH 3, and the precipitate was extracted with ethyl acetate (3 × 30 ml). The combined organic layer was washed with brine, dried over anhydrous sodium sulfate, filtered, and evaporated under reduced pressure to afford a white powder. The crude acid thus obtained was dissolved in dry dichloromethane (20 ml), and DCC (0.74 g, 3.6 mmol) was added at 0 ℃ under nitrogen. Thereafter, a solution of HOBt (0.49 g, 3.6 mmol) in dimethyl formamide (DMF) (5 ml) was added, followed by addition of boc-piperazine (0.56 g, 3 mmol). The reaction mixture was stirred at room temperature overnight. After completion of the reaction, precipitated DCU was filtered off, and the filtrate was diluted with dichloromethane and washed with a saturated bicarbonate solution (2 × 30 ml) and a 10% citric acid solution (2 × 30 ml). The organic layer was then washed with brine (2 × 30 ml), dried over anhydrous sodium sulfate, filtered, and concentrated to a residue. The crude residue was purified by silica gel column chromatography using ethyl acetate hexane (3:7) as the eluent to afford the desired product **3a** and **3b** as a white solid.

### Synthesis of compound 4a

The compound **3a** (0.5 g, 1.34 mmol) was dissolved in a solution of 4 M HCl in anhydrous dioxane at room temperature with vigorous stirring. The reaction mixture was stirred for 2 h at room temperature. After completion of the reaction, dioxane was removed by evaporation at reduced pressure, and the residue was precipitated by adding cold diethyl ether. The precipitate was filtered off, washed with cold diethyl ether, and dried in a vacuum desiccator to afford a white powder as the hydrochloride salt of compound **4a** (0.38 g, 92% yield).

### Synthesis of compound 4b

To a solution of compound **4a** (0.31 g, 1 mmol) in dry pyridine (0.8 ml) was added acetic anhydride (1.02 ml, 10 mmol), and the mixture was stirred overnight at room temperature. After completion of the reaction, the reaction mixture was diluted with water (20 ml) and extracted with ethyl acetate (3 × 30 ml). The combined organic layer was washed with brine (2 × 30 ml), dried over anhydrous sodium sulfate, filtered, and evaporated to dryness. The oily residue was purified by silica gel column chromatography, using ethyl acetate hexane (3:7) as the eluent, to produce compound **4b** (0.26 g, 82%) as a colorless oil.

### Synthesis of compound 4c

Benzyl bromide (0.07 ml, 0.6 mmol) was added to a suspension of compound **4a** (0.136 g, 0.5 mmol) and anhydrous K_2_CO_3_ (0.166 g, 1.2 mmol) in dichloromethane (3 ml). The reaction mixture was stirred overnight at room temperature. Water (30 ml) was then added to the reaction mixture, and the mixture was extracted with dichloromethane (3 × 30 ml). The combined organic layer was washed with brine, dried over anhydrous sodium sulfate, filtered, and evaporated under reduced pressure to give a gummy residue, which was purified by silica gel column chromatography, using ethyl acetate hexane (3:7) as eluent, to afford compound **4c** as a colorless oil (0.123 g, 68%).

### Synthesis of compound 6d

A solution of HOBt (0.233 g, 1.73 mmol) in DMF (2 ml) was slowly added to a mixture of 2-methylthiazole-4-carboxylic acid (0.206 g, 1.44 mmol) and DCC (0.356 g, 1.73 mmol) in dichloromethane (18 ml) at 0 ℃. Thereafter, a solution of compound **6a** (0.41 g, 1.34 mmol) in DMF (2 ml) was added, followed by the addition of DIPEA (0.375 ml, 2.16 mmol). The reaction mixture was stirred for 12 h at room temperature. After completion of the reaction, the mixture was cooled for 2 h at 0 ℃ and the precipitated DCU was filtered off. The filtrate was then washed with 1 N HCl (3 × 30 ml), 10% aqueous sodium bicarbonate (3 × 30 ml) and brine (2 × 30 ml). The organic layer was dried over anhydrous sodium sulfate, filtered, and evaporated to afford a crude residue, which was purified by silica gel column chromatography, using ethyl acetate hexane (3:7) as the eluent, to compound **6d** (0.45 g, 84%) as a white solid.

### Synthesis of compound 5

A solution of sodium borohydride (0.57 g, 15 mmol, 4 equiv) in methanol (4 ml) was added slowly over 15 min to a solution of compound **2a** (0.87 g, 3.75 mmol) in tetrahydrofuran (16 ml) at 50 ℃ under nitrogen. The reaction mixture was refluxed for an additional 1 h and cooled to room temperature, and cold water (10 ml) was added slowly. The volatiles were removed under reduced pressure, and the aqueous residue was extracted with ethyl acetate (3 × 30 ml) and washed with brine (2 × 30 ml). The organic layer was dried over anhydrous sodium sulfate, filtered, and evaporated under reduced pressure. The residue was purified by silica gel column chromatography, using ethyl acetate hexane (2:8) as the eluent, to afford compound **5** as a white solid (0.67 g, 93%).

### Synthesis of compound 6

To a stirred solution of compound **5** (0.58 g, 3 mmol) in THF (30 ml) was added triethylamine (1.25 ml, 9 mmol), followed by the addition of mesyl chloride (1.2 ml, 18 mmol) at 0 ℃. The reaction mixture was stirred at 0 ℃ for 30 min, saturated aq. sodium bicarbonate (30 ml) was added, and the aqueous layer was extracted with ethyl acetate (3 × 50 ml). The combined organic layer was washed with brine, dried over anhydrous sodium sulfate, and evaporated under reduced pressure. The crude product obtained was dissolved in DMF (15 ml), and boc-piperazine (0.67 g, 3.6 mmol) was added, followed by the addition of anhydrous potassium carbonate (0.83 g, 6 mmol). The reaction mixture was stirred for 5 h at 80 ℃. After completion of the reaction, the reaction mixture was poured into cold water (50 ml) and extracted with ethyl acetate (50 ml × 3). The combined organic layer was washed with brine, dried over anhydrous sodium sulfate, filtered, and concentrated under reduced pressure. The crude residue was purified by silica gel column chromatography, using ethyl acetate hexane (3:7) as the eluent, to afford compound **6** as a white solid (0.87 g, 81%).

### Synthesis of compound 7a

The method for synthesis of this compound was same as that for compound **4a**, but the starting material used in this case was compound **6**. Compound **7a** was isolated after purification as a white solid (0.072 g, 93%).

### Synthesis of compound 7b

Dry THF (10 ml) was added dropwise under N_2_ atmosphere to an ice cooled RBF containing LiAlH_4_ (0.114 g, 3.0 mmol, 3 equiv.) at 0 °C. After completion of the THF addition, compound **6** (1 mmol) in dry THF was added slowly over 30 min at 0 °C, and the resulting mixture was stirred at room temperature for 2 h. After completion of the reaction, ice was added to the resulting reaction mixture and the precipitate was filtered off. The filter cake was washed with diethylether (3 × 20 ml) and then with ethyl acetate (3 × 10 ml). The combined organic phase was dried over anhydrous sodium sulfate, filtered and evaporated under vacuum to produce light yellow oil. The residue was purified by silica gel column chromatography using methanol chloroform (1:19) as eluent to give compound 7b colorless oil (0.109 g, 40%).

### Synthesis of compound 7c

The method for synthesis of this compound was same as that for compound **4b** but the starting material used in this case was compound **7a**. This compound was isolated after purification as a colorless oil (0.058 g, 74%).

### Synthesis of compound 7d

The method for synthesis of this compound was same as that for compound **7b** but the starting material used in this case was compound **7c**. This compound was isolated after purification as light yellow oil (0.076 g, 53%).

### Synthesis of compound 7e

The method for synthesis of this compound was same as that for compound **4c** but the starting material used in this case was compound **7a**. This compound was isolated after purification as white solid (0.07 g, 71%).

### In-vitro translation

The inhibition effect of the compounds (and of chloramphenicol as the reference compound) on *M. smegmatis* ribosomes was tested in a bacterial coupled transcription/translation assay, in which the expression of luciferase gene was measured. The luciferase gene was inserted into the plasmid downstream from the T7 RNA polymerase promotor. The reaction mixture contained 160 mM HEPES-KOH (pH 7.5), 6.5% polyethylene glycol 8000, 0.074 mg/ml tyrosine, 1.3 mM ATP, 0.86 mM for CTP, GTP and UTP, 208 mM potassium glutamate, 83 mM creatine phosphate, 28 mM ammonium acetate, 0.663 mM cAMP, 1.8 mM DTT, 0.036 mg/ml folinic acid, 0.174 mg/ml *Escherichia coli* tRNA mix, 1 mM of each amino acid, 0.25 mg/ml creatine kinase, 0.044 mg/ml T7 RNA polymerase, 25% V/V S30 *M. smegmatis* cell-free extract, 7 ng/µl luciferase-encoding plasmid and the compound to be tested at a final concentration of 1 mM. Molecules that showed significant inhibition at a concentration of 1 mM were further tested in concentrations ranging from 6 nM to 1 mM. The reaction mixture was incubated at 37 ℃ for 1 h, and the reaction was terminated by the addition of erythromycin at a final concentration of 8 µM. To quantify the reaction products, luciferin assay reagent (LAR, Promega) was added at 5:3 (luciferase: reaction mix) volume ratio, and luminescence was measured in a plate reader (BioTek SYNERGY H1). The results were plotted (compound concentration vs. luminescence intensity), and IC_50_ values were calculated.

### S30 extract

*M. smegmatis* was cultured by using established protocols with minor modifications [26, 27] *M. smegmatis* cells were grown in LB medium (5 g yeast extract, 5 g NaCl, 10 g/L Tryptone) with continuous shaking (200 rpm) at 30 ℃ to OD 0.6 to 0.8. 0.04% (w/v). Tween 80 was added to LB to avoid clumping. The cells were harvested by centrifugation and washed twice in 200 ml buffer A (10 mM HEPES-NaOH [pH 7.4], 60 mM K-glutamate, 14 mM MgCl_2_), and fresh 7 mM β-mercaptoethanol. The cells were resuspended in buffer A to 0.5 g/ml and lysed with a high-pressure homogenizer at 20,000 psi. The extract was centrifuged at 30,000 RCF for 30 min, and the supernatant was removed to a new vial twice. The supernatant incubated at 37 ℃ for 1.5 h and dialysed for 2 h at 4 ℃ against buffer A. The *M. smegmatis* S30 extract was aliquoted and kept frozen.

### In silico molecular docking

Molecular docking was performed as described [9] by using AutoDock 4.2 [10, 11] to estimate the binding free energy (ΔG*bind*) and the poses of the investigated compounds in relation to the receptor. The PTC receptor for simulations was derived from the Cartesian coordinates of the large ribosomal subunit (50S) of *Staphylococcus aureus* (PDBID 4WCE [28]). The virtual screening protocol was conducted through the Raccoon implementation (http://autodock.scripps.edu/resources/raccoon), in which the receptor and ligand molecules were preprocessed for docking. The docking grid was set with 126 points in each dimension, and the default spacing was 0.375 Å. The obtained grid map was centered with respect to the receptor. Free energy calculations and conformational sampling of the ligands were then carried out using the Lamarckian genetic algorithm (LGA), with an initial population size of 150 individuals, 2,500,000 free energy evaluations and 27,000 LGA generations. Clustering of the results was performed by root mean-square deviation (RMSD) calculations for the obtained poses of the ligands, with a constant tolerance of 2.0 Å. Further analyses of the results were performed using default AutoDock VS tutorial scripts along with several in-house written scripts.

#### Choosing the dataset for the model

As previously described [9], transverse relaxation NMR spectroscopy adapted for fragment screening selected 2-phenylthiazole as a molecular scaffold. Importantly, our data is labeled, i.e., every molecule in the dataset (860) is assigned to a binding value to the RNA target. These binding values were obtained by virtual screening. Molecules that appeared twice with the same name (as a result of different charge or oxidation state) were deleted.

### Decision tree

A decision tree is designed to aid in planning new potential inhibitory molecules by identifying essential features that differentiate molecules with high binding scores from molecules with low binding scores. We have divided the data (stratified split) into two groups, binders and non-binders, on the basis of their binding values. The top 2.5% percentile of the docking scores (≤ − 13.2) was determined as a threshold. For this model, all 204 chemical descriptors of the small molecules extracted using RDkit were used. After removing zero-variance features, features were further reduced using ‘Forward Selection’ wrapper method on the decision tree model, resulting in a three features dataset. The decision tree classifier was trained using tenfold cross-validation on the training data, to produce 100% accuracy on the test sets.

### Regression model

The Lasso regressor is a linear regression with regularization (the magnitude of the penalty is an adjustable hyperparameter), which has an embedded feature selection property by penalizing to decrease degrees of model complexity. In Lasso, inessential feature weights are pushed to zero, and are thus eliminated from the model [29]. The Lasso model loss function is:

$${\varvec{Loss}} = { }\frac{1}{{\varvec{n}}}{ }\sum \left( {{ }{\mathbf{y}}{ }{-}{ }{\hat{\mathbf{y}}}{ }} \right)^{2} + {\varvec{\alpha}}\sum { }\left| {\varvec{\theta}} \right|$$

where the sum of mean squared errors (y − ŷ)^2^ is computed with L1 regularization, which comprises the sum of all weighs (Ɵ) multiplied with the penalty term (α). Regularization allows to remove variable attaining zero, and thereby removing irrelevant features from the model [30].

The chemical descriptors used for the regressor are: NHOHCount, TPSA, NOCount, EState_VSA8, PEOE_VSA1, SMR_VSA7, HallKierAlpha, EState_VSA7, NumHDonors, VSA_EState8, EState_VSA9, SlogP_VSA8, SlogP_VSA11, VSA_EState2, EState_VSA5, VSA_EState9, Chi3v, EState_VSA6, VSA_EState5, MolLogP, BalabanJ, SMR_VSA2, PEOE_VSA2, PEOE_VSA3, SlogP_VSA2, SlogP_VSA10, EState_VSA4, PEOE_VSA10, PEOE_VSA13, PEOE_VSA7, SlogP_VSA4, PEOE_VSA11. The diagonal correlation matrix of the 32 features is presented in Additional file 1: Fig. S1.

### Feature engineering

First, all features were normalized to standard scores (z-score). The data was divided into training set (80%) and a test set (20%). The features selection and the hyperparameter tuning were evaluated by the mean of the test groups of tenfold cross validation. A summary of the hyper-parameters tuning for all models is presented in Additional file 1: Table S6. The approaches used to select the optimal set of features are: (1) Lasso regression, (2) removal of features with zero-variance (below 0.03), (3) removal of features that are highly collinear to other features (Pearson correlation above 0.9), (4) removal of features with no correlation to the binding score (the label), and (5) forward/backward features selection that allow choosing the most significant features for prediction, by iterative selection and evaluation of each feature, one at a time. In backward feature selection, the model is fit with all the features, then the least significant feature is examined against p-value, and if it is high (> 0.05), this feature is removed from the model. After several iterations of fitting to the model and removal of features, the model remains only with significant 32 features. A list of selected features is presented in and their Pearson correlation in Additional file 1: Fig. S1.

#### Training the model

Following the selection of the features and the hyperparameters, the model was lunched over all the training data, and the weights and bias were extracted. The final model was evaluated over the test data. The importance of the features was estimated by the values of linear regression coefficients and by the global SHAP value of each feature.

### CNN architecture SMILES representation of the input data

Since SMILES constitutes an indexed sequential representation of the molecules, a pre-processing step was required to convert the "words" into matrices of numbers that the computer can process. The SMILES strings were converted into matrices, each containing 42-bit vectors (inspired by the design of Hirohara et al. [17], where 21 bits were used for an atom and chemical description such as degree of unsaturation, formal charge, total valence, aromaticity and ring content, chirality, and hybridization, using RDKit, and 21 bits for structural information, Additional file 1: Table S3). Zero-padding vectors were added to the bottom of each matrix, in order to standardize the matrix size. Matrices of 240 × 42 were then used as input into the CNN. The dataset containing 791 molecules was split into 80% of the molecules (632) training set, and 20% (159) test set. Fivefold cross-validation training (using only the training data) was performed for model optimization and hyperparameter tuning. The test MAE was averaged over the five sub-groups for each, and the model with the lowest MAE was chosen.

The CNN network consists of two convolutional layers and two fully connected layers separated by global max-pooling. Rectified activation function (Rectified Linear Unit, ReLU [31]) was applied on the convolution results in the hidden layers and after the first fully connected operation. As we aimed to solve a regression type of problem, no activation function was used for the output layer (after the second fully connected layer). Batch normalization is applied after each computational layer, and then average pooling was applied on the matrices. All weights were initialized by a normal distribution with a mean of 0 and a standard deviation of 0.01. The model was trained upon mini-batch (32 instances each) gradient descent for 1200 epochs. Optimization was achieved using ADAM [32] with a learning rate of 5*10^–5^. The weights of the best test loss achieved were saved as the final model.

The output from the global max-pooling layer was extracted for the entire dataset and continued to the fully connected layer. Data was normalized for each filter separately and converted into z-scores for normalization. Molecules' pixels with Z-scores larger than 2.58 (i.e., 99% percentile) were chosen, and the receptive field was calculated for these molecules; meaning that, from the one pixel that remains in each filter, we took only pixels that had values of the top 1% in a normal distribution after normalizing the data. Therefore, these pixels' receptive field represents the most significant motif contributor to the score.

### CNN architecture; pictorial representation of the input data

#### Preprocessing of data

The dataset contained 791 color images of molecular graphs (783 × 1316 × 3), each assigned to binding score of docking to the RNA target. For pre-processing the foreground data was dilated for each image, then cropped to reduce background pixels, which resized the images to 775 × 775 × 3. Next, images were resized to 50 × 50 × 3. The images' pixel values were normalized into the range [0,1]]. In addition, the color images were converted to gray scale and to binary images for different experiments and then the same pre-processing was applied (Additional file 1: Fig. S2). The data was divided into a training set (80%, 632 images) and a test set (20%, 159 images).

#### Designing the model and training

For model optimization and hyperparameter tuning, we used a fivefold cross-validation. The test MAE was averaged over the five sub-groups for each, and the model with the lowest MAE was chosen. The processed pixel values of the images were used as input for a CNN. The model contains 2 convolution layers followed by max pooling and 2 dense layers at the end of the network. For the hidden layer, rectified activation function (Rectified Linear Unit, ReLU [33]) was applied on all matrices within hidden layers. As we aimed to solve a regression type of problem, no activation function was used for the output layer, i.e. linear output layer. For the optimization, the ADAM algorithm [32] was used and the loss function was expressed in terms of mean absolute error (MAE). Dropout layer of 20% was added before the final layer. The learning rate was 0.001.

## Supplementary Information

**Additional file 1.** Supplementary information includes additional tables and figures.

## Data Availability

Dataset and code in python are available at https://github.com/csbarak/primroseLightning.

## Abbreviations

RNA: Ribonucleic acid
PTC: Peptidyl transferase center
SAR: Structure–activity relationships
NMR: Nuclear magnetic resonance
CNN: Convolutional neural networks
MAE: Mean average error
SHAP: Shapley additive explanations
SMILES: Simplified Molecular Input Line Entry System
IC-50: Inhibitory concentration of half maximum activity
PDB: Protein data bank
THF: Tetrahydrofuran

## References

[1] Langmuir I (1919). Isomorphism, isosterism and covalence. J Am Chem Soc.

[2] Lavecchia A (2019). Deep learning in drug discovery: opportunities, challenges and future prospects. Drug Discov Today.

[3] Chen H, Engkvist O, Wang Y, Olivecrona M, Blaschke T (2018). The rise of deep learning in drug discovery. Drug Discov Today.

[4] Gawehn E, Hiss JA, Schneider G (2016). Deep learning in drug discovery. Mol Inform.

[5] Patel L, Shukla T, Huang X, Ussery DW, Wang S (2020). Machine learning methods in drug discovery. Molecules.

[6] Musella S, Verna G, Fasano A, Di Micco S (2020). New perspectives of machine learning in drug discovery. Curr Med Chem.

[7] Hussain W, Rasool N, Khan YD (2020). Insights into machine learning-based approaches for virtual screening in drug discovery: existing strategies and streamlining through FP-CADD. Curr Drug Discov Technol.

[8] Vamathevan J (2019). Applications of machine learning in drug discovery and development. Nat Rev Drug Discov.

[9] Tam B (2019). Discovery of small-molecule inhibitors targeting the ribosomal peptidyl transferase center (PTC) of *M. tuberculosis*. Chem Sci.

[10] Goodsell DS, Morris GM, Olson AJ (1996). Automated docking of flexible ligands: applications of AutoDock. J Mol Recognit.

[11] Irwin JJ, Shoichet BK (2005). ZINC–a free database of commercially available compounds for virtual screening. J Chem Inf Model.

[12] RDKit: open-source cheminformatics software. (https://www.rdkit.org/)

[13] Rodriguez-Perez R, Bajorath J (2020). Interpretation of compound activity predictions from complex machine learning models using local approximations and shapley values. J Med Chem.

[14] Lundberg SM, Lee SI (2017) In 31st Conference on Neural Information Processing Systems (NIPS 2017) (Long Beach, CA).

[15] Kier LB, Hall LH (1991) The molecular connectivity chi indices and kappa shape indices in structure-property modeling. (Wiley-VCH)

[16] Weininger D (1988). a chemical language and information system. J Chem Inf Comput Sci.

[17] Hirohara M, Saito Y, Koda Y, Sato K, Sakakibara Y (2018). Convolutional neural network based on SMILES representation of compounds for detecting chemical motif. BMC Bioinform.

[18] Luo W, Li Y, Urtasun R, Zemel R (2017) Understanding the effective receptive field in deep convolutional neural networks. arXiv:1701.04128

[19] Araujo A, Norris W, Sim J (2019). Computing receptive fields of convolutional neural networks. Distill.

[20] Matzov D (2017). Structural insights of lincosamides targeting the ribosome of *Staphylococcus aureus*. Nucleic Acids Res.

[21] Hentschel J (2017). The complete structure of the *Mycobacterium smegmatis* 70S ribosome. Cell Rep.

[22] Demšar J (2013). Orange: data mining toolbox in Python. J Mach Learn Res.

[23] Prasanna S, Doerksen RJ (2009). Topological polar surface area: a useful descriptor in 2D-QSAR. Curr Med Chem.

[24] Hall LH, Mohney B, Kier LB (1991). The electrotopological state—structure information at the atomic level for molecular graphs. J Chem Inf Comp Sci.

[25] Labute P (2000). A widely applicable set of descriptors. J Mol Graph Model.

[26] Srivastava A (2016). Reconstitution of protein translation of Mycobacterium reveals functional conservation and divergence with the gram-negative bacterium *Escherichia coli*. PLoS ONE.

[27] Liu DV, Zawada JF, Swartz JR (2005). Streamlining *Escherichia coli* S30 extract preparation for economical cell-free protein synthesis. Biotechnol Prog.

[28] Eyal Z (2015). Structural insights into species-specific features of the ribosome from the pathogen *Staphylococcus aureus*. Proc Natl Acad Sci USA.

[29] Ranstam J, Cook JA (2018). LASSO regression. J Br Surg.

[30] Tibshirani R (1996). Regression shrinkage and selection via the lasso. J Roy Stat Soc.

[31] Glorot X, Bordes A, Bengio Y In Proceedings of the 14th International Conference on Artificial Intelligence and Statistics (AISTATS)

[32] Kingma DP, Ba J (2014) Adam: A Method for Stochastic Optimization

[33] Glorot X, Bordes A, Benjio Y. In Proceedings of the 14th International Conference on Artificial Intelligence and Statistics 315–323.

