# Supplementary material for: Machine learning approaches to optimize small-molecule inhibitors for RNA targeting
Source: J Cheminform. 2022 Feb 2;14:4. doi: 10.1186/s13321-022-00583-x (PMC8811966; doi:10.1186/s13321-022-00583-x)
Supplement: Supplementary file 1 — Additional file 1. Supplementary information includes additional tables and figures. [file 13321_2022_583_MOESM1_ESM.docx]

*Supporting information:* **Machine learning approaches to optimize small-molecule inhibitors for RNA targeting**

Hadar Grimberg , Vinay S. Tiwari, Benjamin Tam, Lihi Gur-Arie, Daniela Gingold, Lea Polachek, Barak Akabayov^1^
Department of Chemistry and Data Science Research Center, Ben-Gurion University of the Negev, Beer-Sheva 8410501, Israel
Correspondence should be addressed to B.A (akabayov@bgu.ac.il)

**Supplementary Table S1.** Summary of various regression models for prediction of binding of small molecules to the ribosomal PTC.

|  | MAE | MAE Variance | MAE best 10% | MSE | MSE Variance | MSE best 10% | R_2_ |
| --- | --- | --- | --- | --- | --- | --- | --- |
| Ridge regression | 0.579 | 0.101 | 1.418 | 7.546 | 333.072 | 61.904 | NA |
| Lasso regression | 0.377 | 0.001 | 0.415 | 0.283 | 0.008 | 0.287 | 0.913 |
| Gradient boosting loss- ls | 0.418 | 0.002 | 0.581 | 0.338 | 0.017 | 0.739 | 0.898 |
| Gradient boosting loss-lad | 0.445 | 0.004 | 0.761 | 0.414 | 0.032 | 1.361 | 0.877 |
| Neural Network | 0.414 | 0.002 | 0.565 | 0.323 | 0.016 | 0.701 | 0.900 |
| XGboost | 0.413 | 0.002 | 0.567 | 0.323 | 0.010 | 0.661 | 0.902 |
| Adaboost Regressor Linear | 0.574 | 0.002 | 1.030 | 0.610 | 0.020 | 1.731 | 0.813 |
| Adaboost Regressor Squared | 0.576 | 0.001 | 1.036 | 0.599 | 0.014 | 1.645 | 0.817 |
| Decision tree | 0.515 | 0.004 | 0.826 | 0.544 | 0.054 | 1.481 | 0.832 |
| Adaboost-RF | 0.467 | 0.003 | 0.841 | 0.392 | 0.024 | 1.184 | 0.883 |
| BayesianRidge | 0.387 | 0.002 | 0.482 | 0.287 | 0.013 | 0.542 | 0.911 |
| SVR | 1.067 | 0.007 | 2.463 | 2.500 | 0.113 | 7.904 | 0.248 |
| RF mse | 0.403 | 0.003 | 0.595 | 0.322 | 0.021 | 0.881 | 0.902 |

**Supplementary Table S2.** Representation of molecular data by SMILES

| Atom |  | 21 |
| --- | --- | --- |
| Atom type | H, C, O, N, or others | 5 |
| NumHs | Total number of H atoms attached to it | 1 |
| Degree | Its degree of unsaturation | 1 |
| Charge | Its formal charge | 1 |
| Valence | Its total valence | 1 |
| Ring | Whether it is included in a ring | 1 |
| Aromaticity | Whether it is included in an aromatic structure | 1 |
| Chirality | R, S, or others | 3 |
| Hybridization | *s*, *sp*, *sp*^2^, *sp*^3^, *sp*^3^*d*, *sp*^3^*d*^2^, or others | 7 |
| SMILES original symbol |  | 21 |
| ( | Branch start | 1 |
| ) | Branch end | 1 |
| [ | Atom or atom group start | 1 |
| ] | Atom or atom group end | 1 |
| . | Ionic bond | 1 |
| : | Aromatic bond | 1 |
| = | Double bond | 1 |
| # | Triple bond | 1 |
| \ | cis | 1 |
| / | trans | 1 |
| @ | Chirality (above or below) | 1 |
| + | Cation (positive ion) | 1 |
| - | Anion (negative ion) | 1 |
| Ion charge | Numbers show ionic charge (2-7) | 6 |
| Start | Numbers show ring start | 1 |
| End | Numbers show ring end | 1 |

**Supplementary Table S3a.** Essential filters of CNN

| **Inhibitor Filter** | | | |
| --- | --- | --- | --- |
| Filter 57 | 19944337 | 5441870 |  |
| **Non-Inhibitor Filter** | | | |
| Filter 1 | 70840402 | 50308763 |  |
| Filter 9 |   158873 | 49857638 |  |
| Filter 17 | 40999569 | 110944 |  |
| Filter 22 |   70842966 |   52859414 |  |
| Filter 24 |   43392655 | 43392661 |  |
| Filter 28 | 110944 | 40999615 |  |
| Filter 40 | 73592 | 2569099 |  |
| Filter 52 | 36827739 | 49633851 |  |
| Filter 62 | 5177788 | 2647760 |  |

**Supplementary Table S3b.** Molecular motifs in SMILES, effect and statistics of CNN filters on target binding.

| Filter # | Sequence motif found (SMILES) | Effect on prediction | Distribution of this feature |
| --- | --- | --- | --- |
| 57 | N:C:1C([H])([H])N(C([H]) N:C:1C([H])([H])N1C([H]) | High binding score | 17/22 (top 2.8%) molecules with score above 13 contain this motif. |
| 9 | [H]C(=O)C1:N:C(C2:C([H]) C([H])([H])C1:N:C(C2:C([H])  O[C@]([H])(C1:N:C(C2:C([H]) [C@@]([H])(C1:N:C(C2:C([H]) N([H])C(=O)C1:N:C(C2:C([H]) [H]C(=O)C1:N:C(C2:C(Cl):C | Low binding score | This sequence is found in 59 molecules with binding score ranges from 5.37 to 10.21. |
| 40 | Cl, I, F or Br | Low / medium binding score | These atoms are found in 316 molecules. 197 of 316 molecules (62.3% of the total 316) had a binding value below the median, -10.15, or worse (about 50% of all molecules with a below median score contain atoms found by this filter). |
| 52 | ([H])([H])C1:N:C(C2:C([H]):C | Low binding score | 39 of 791 molecules (4.9% of all the molecules) contain this sequence; binding score range between -5.37 and -10.21; 37 molecules (94.8% of 39) contain O. |
| 62 | ([H])Cl  C(=O)Cl  (F)(F)F  C:1Cl  ([H])I  ([H])Br | Low/ medium binding score | 51 of 791 molecules (6.4% of total). Binding score ranged from -5.65 to -12.86; 35 of the 51 molecules (68.6%) had binding score below the median (out of 395, 8.9%). |
| 1 | [H]C1:S:C(C2:C(Cl):C [H]C1:S:C(C2:C(F):C([H]) 'C([H]):C(C2:N:C(C([H])([H]) N:C([H]):C(C2:N:C(C([H])([H] | Low/ medium binding score | This motif is found in 34 of the molecules. Binding score ranged from -5.31 to -11.22; 23 of 395 molecules (about 67.6% of the 34) have binding score below the median (out of 395, 5.8%). |
| 17 | ([H]):C([H]):C(C(F)(F)F):C:2[H] ([H]):C([H]):C(OC(F)(F)F):C([H]) ([H]):C([H]):C:2C(F)(F)F):N:C C(Cl):C([H]):C(C(F)(F)F):C | Low binding score | Found in 5 of the molecules. Binding score ranges between -6.11 to -9.55. All molecules have binding score values under the average and the median. |
| 22 | C(=O)C1:N:C(C2:C([H]):C(Cl) ([H])C1:N:C(C2:C([H]):C(Cl) ([H])(C1:N:C(C2:C([H]):C([H]) ([H]):C(C2:N:C(C3:C([H]):C([H]) | Low binding score | Found in 10 of the molecules. Binding score ranges between -5.59 to -7.7. All of them have binding score below the average and below the median. |
| 24 | S:C(C2:C([H]):C(F):C(F):C [H]C1:N:C([H]):C(C2:N:C(C([H]) | Low/ medium binding score | Found in 10 of the molecules. Binding score ranges between -9.15 to -10.51. (about 90 % of the 10) have binding score below the median (out of 395, 2.3%). |
| 28 | [H]C1:S:C(C2:C(Cl):C([H]) [H]C1:S:C(C2:C(F):C([H]):C [H]C1:S:C(C2:C(Cl):C([H]) (=O)C1:N:C(C2:C(Cl):C([H]):C | Low/ medium binding score | Found in 28 of the molecules. Binding score ranged between -6.11 to -11.22. 17 (about 65.4% of the 26) have binding score below the median (out of 395, 4.3%). |

**Supplementary Table S4.** Important molecular motifs in SMILES from filter 57 for strong binding to the ribosomal PTC

| # | Name/SMILES | Zinc # | Structure | Binding score |
| --- | --- | --- | --- | --- |
| 1 | [H]C1:S:C(C2:C([H]):C([H]):C([H]):C([H]):C:2[H]):N:C:1C([H])([H])N(C([H])([H])[H])C([H])([H])C([H])([H])[N+]([H])([H])[H] | ZINC54418570 |  | -14.5 |
| 2 | [H]C1:S:C(C2:C([H]):C([H]):C([H]):C([H]):C:2Cl):N:C:1C([H])([H])N(C([H])([H])[H])C([H])([H])C([H])([H])[N+]([H])([H])[H] | ZINC54418918 |  | -14.84 |
| 3 | [H]C1:S:C(C2:C([H]):C([H]):C(C([H])([H])[H]):C([H]):C:2[H]):N:C:1C([H])([H])N(C([H])([H])[H])C([H])([H])C([H])([H])[N+]([H])([H])[H] | ZINC54418966 |  | -15.22 |
| 4 | [H]C1:S:C(C2:C([H]):C([H]):C([H]):C([H]):C:2[H]):N:C:1C([H])([H])N(C([H])([H])[H])C([H])([H])C([H])([H])[N+]([H])([H])C([H])([H])[H] | ZINC70304164 |  | -14.05 |
| 5 | [H]C1:S:C(C2:C([H]):C([H]):C([H]):C([H]):C:2[H]):N:C:1C([H])([H])N1C([H])([H])C([H])([H])[N+]([H])([H])C([H])([H])C1([H])[H] | ZINC19944337 |  | -14.26 |
| 6 | [H]C1:S:C(C2:C([H]):C([H]):C([H]):C([H]):C:2[H]):N:C:1C([H])([H])[N+]12C([H])([H])C([H])([H])N(C([H])([H])C1([H])[H])C([H])([H])C2([H])[H] | ZINC12546573 |  | -13.85 |
| 7 | [H]C1:S:C(C2:C([H]):C([H]):C([H]):C([H]):C:2[H]):N:C:1C([H])([H])N1C([H])([H])C([H])([N+]([H])([H])[H])C1([H])[H] | ZINC87590125 |  | -14.93 |
| 8 | [H]C1:S:C(C2:C([H]):C([H]):C([H]):C([H]):C:2Cl):N:C:1C([H])([H])N1C([H])([H])C([H])([H])[N+]([H])(C([H])([H])[H])C([H])([H])C1([H])[H] | ZINC23253647 |  | -15.15 |
| 9 | [H]C1:S:C(C2:C([H]):C([H]):C([H]):C([H]):C:2Cl):N:C:1C([H])([H])N1C([H])([H])C([H])([H])[N+]([H])([H])C([H])([H])C1([H])[H] | ZINC20221711 |  | -14.18 |
| 10 | [H]C1:S:C(C2:C([H]):C([H]):C([H]):C([H]):C:2Cl):N:C:1C([H])([H])N(C([H])([H])[H])C([H])([H])C([H])([H])[N+]([H])([H])C([H])([H])[H] | ZINC70304038 |  | -14.08 |
| 11 | [H]C1:S:C(C2:C([H]):C([H]):C(C([H])([H])[H]):C([H]):C:2[H]):N:C:1C([H])([H])N(C([H])([H])[H])C([H])([H])C([H])([H])[N+]([H])([H])C([H])([H])[H] | ZINC70304040 |  | -13.27 |
| 12 | [H]C1:S:C(C2:C([H]):C([H]):C(C([H])([H])[H]):C([H]):C:2[H]):N:C:1C([H])([H])N1C([H])([H])C([H])([H])[N+]([H])([H])C([H])([H])C1([H])[H] | ZINC19944344 |  | -14.6 |
| 13 | [H]C1:S:C(C2:C([H]):C([H]):C(C([H])([H])[H]):C([H]):C:2[H]):N:C:1C([H])([H])N1C([H])([H])C([H])([N+]([H])([H])[H])C1([H])[H] | ZINC87590112 |  | -14.73 |
| 14 | [H]C1:S:C(C2:C([H]):C([H]):C([H]):C([H]):C:2[H]):N:C:1C([H])([H])N1C([H])([H])C([H])([H])[N+]([H])(C([H])([H])C([H])([H])C([H])([H])[H])C([H])([H])C1([H])[H] | ZINC23253563 |  | -14.04 |
| 15 | [H]C1:S:C(C2:C([H]):C([H]):C([H]):C([H]):C:2Cl):N:C:1C([H])([H])N1C([H])([H])C([H])([N+]([H])([H])[H])C1([H])[H] | ZINC87589974 |  | -14.66 |
| 16 | [H]C1:S:C(C2:C([H]):C([H]):C([H]):C([H]):C:2[H]):N:C:1C([H])([H])N(C([H])([H])[H])C1([H])C([H])([H])[N+]([H])([H])C1([H])[H] | ZINC90290472 |  | -14.37 |
| 17 | [H]C1:S:C(C2:C([H]):C([H]):C([H]):C([H]):C:2[H]):N:C:1C([H])([H])N(C([H])([H])C([H])([H])[H])C1([H])C([H])([H])[N+]([H])([H])C1([H])[H] | ZINC90292053 |  | -14.28 |

**Supplementary Table S5.** Means and (SDs) of two out of three most important chemical features (found by the regression model) for substructures revealed by CNN filters.

| Filter | 57 | 1 | 9 | 17 | 22 | 24 | 28 | 40 | 52 | 62 |
| --- | --- | --- | --- | --- | --- | --- | --- | --- | --- | --- |
| TPSA | 35.09 (5.41) | 5.25 (10.38) | 20.47 (9.52) | 3.50 (7.68) | 19.22 (8.25) | 3.86 (10.2) | 3.55 (12.3) | 0 (0) | 14.10 (0) | 0.71 (3.48) |
| N, O count | 1.94 (0.24) | 0.33 (0.62) | 1.36 (0.52) | 0.22 (0.44) | 1.30 (0.48) | 0.29 (0.76) | 0.17 (0.58) | 0 (0) | 1.00 (0) | 0.04 (0.2) |
| n* | 17 | 15 | 53 | 9 | 10 | 7 | 12 | 24 | 33 | 24 |

*n= Count of molecules found by the filter (had z-score of 2.58 or above)

**Supplementary Table S6: A summery table of the hyper-parameters tuning for all models.**

| **Model** | **Parameters** | **Tested Options (chosen in bold)** |
| --- | --- | --- |
| Regression |  |  |
|  | Alpha | **0**, 0.1, 0.2, 1 |
| Decision Tree |  |  |
|  | Max depth | 2, **3** |
| CNN SMILES |  |  |
|  | Channels conv layer 1 | **128**, 360 |
|  | Channels conv layer 2 | **64**, 180 |
|  | Kernel size conv layer 1 | (5, 42), **(11, 42)** |
|  | Kernel size conv layer 2 | **(11, 1)**, (19, 1), (49, 1) |
|  | Pool size of averagePooling2D layer 1 | **(5,1)**, (9,1), (19,1) |
|  | Pool size of averagePooling2D layer 2 | **(5,1)**, (17, 1), (33,1) |
|  | Neurons on FC1 | **32**, 48, 60, 70, 80, 90 |
|  | Kernel FC1 l2 regularizer | 0.01, **0.001** |
|  | Bias FC1 l2 regularizer | 0.01, **0.001** |
|  | Learning rate | 0.0005, 0.0001, **0.00005**, 0.00001 |
|  | Batch size | **32**, 70 |
| CNN pictural |  |  |
|  | Batch size | **1**, 16 |
|  | Learning rate | 0.01, **0.001**, 0.0001 |
|  | Channels conv layer 1 | 8, **16**, 32 |
|  | Channels conv layer 2 | 8, 16, **32** |
|  | Neurons on FC1 | **32**, 64 |

**Supplementary Table S7**. Chemical structure of the molecules synthesised and bio-evaluated as inhibitors for *Mycobacterial* ribosomes.

## Supplementary Figures


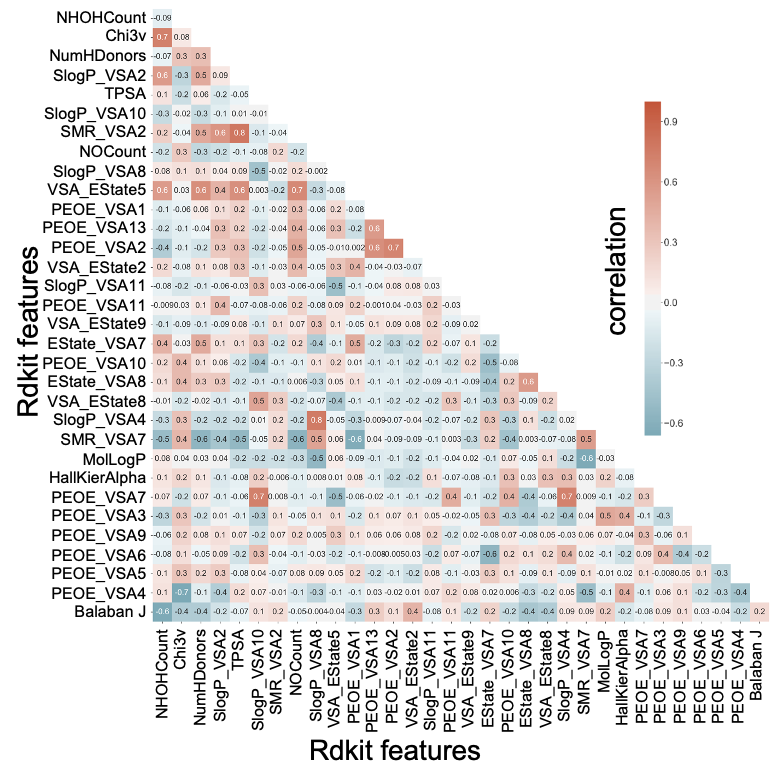


**Supplementary Fig. S1.** Diagonal correlation matrix of the 32 features that were used for the regression model after feature extraction by using RDKit library (RDKit: Open-source cheminformatics; [http://www.rdkit.org](http://www.rdkit.org/)). The features are: NHOHCount, Chi3v, NumHDonors, SlogP_VSA2, TPSA, SlogP_VSA10, SMR_VSA2, NOCount, SlogP_VSA8, VSA_EState5, PEOE_VSA1, PEOE_VSA13, PEOE_VSA2, VSA_EState2, SlogP_VSA11, PEOE_VSA11, VSA_EState9, EState_VSA7, PEOE_VSA10, EState_VSA8, VSA_EState8, SlogP_VSA4, SMR_VSA7, MolLogP, HallKierAlpha, PEOE_VSA7, PEOE_VSA3, PEOE_VSA9, PEOE_VSA6, PEOE_VSA5, PEOE_VSA4, BalabanJ. The Figure was created using Seaborn python library (https://seaborn.pydata.org/ ).


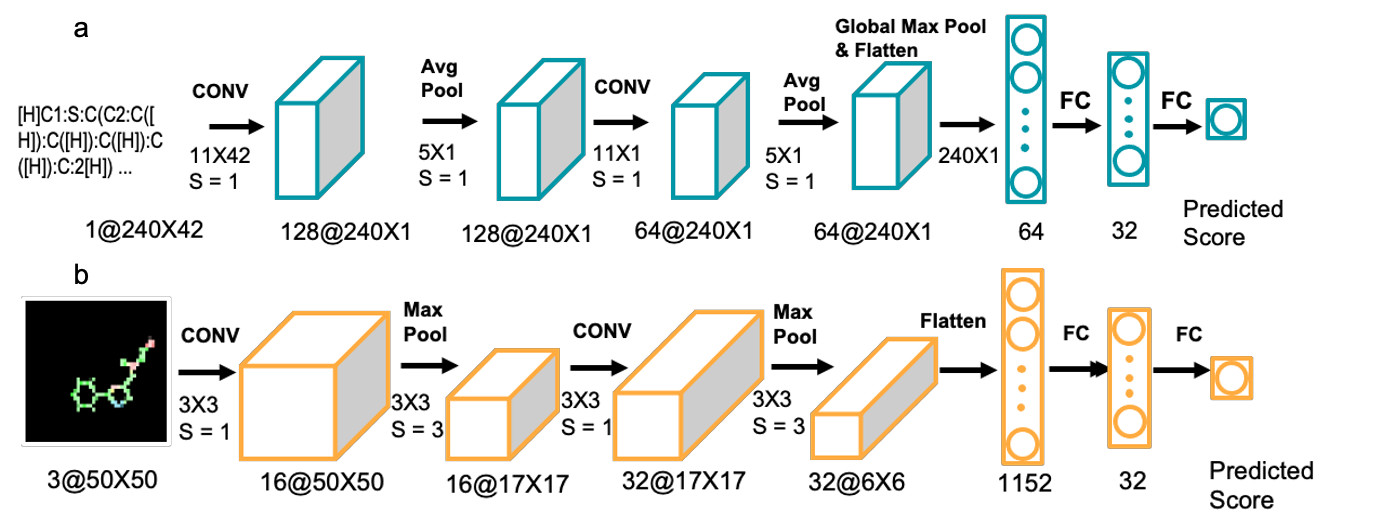


**Supplementary Fig. S2. Architecture of the convolutional neural networks (CNNs) used in this study.** Two CNN architectures were used for the analysis of two types of input data: 1) SMILES strings, and 2) images. The CNN architecture that we built consists of three layers: convolutional (CONV), pooling (Avg/Max Pool) and fully connected (FC).


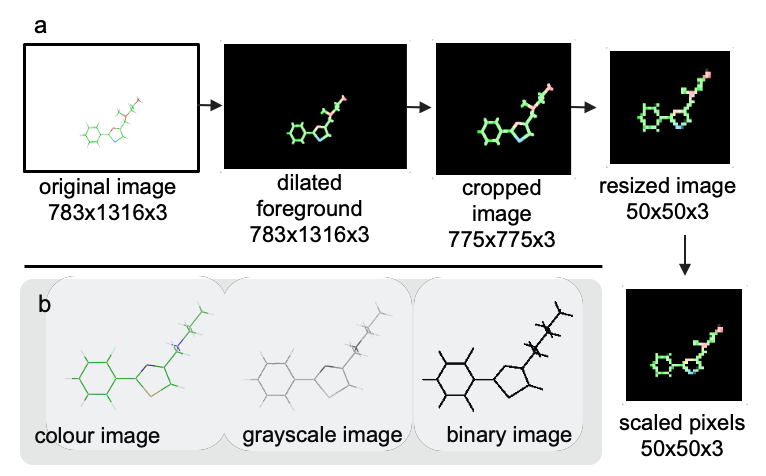


**Supplementary Fig. S3. Image pre-processing for CNN.** (a) The process included converting the images as arrays, dilating foreground for each image, cropping images according to the largest mol in the data, resizing images to 50x50x3, scaling images’ values into the range [0,1]. (b) A sample image in color, grayscale and binary representation.

b3

b2

b1

a

**Supplementary Fig. S4. Learning curves of the loss by CNN.** Progress of loss in three different types of CNN model and image input. A. CNN SMILES. B. CNN images; color, grey scale, and binary. The blue line denote the training curve and the orange line denotes the validation of the CNN algorithm.The network trained on GPU provided by Google Collab ^1^.


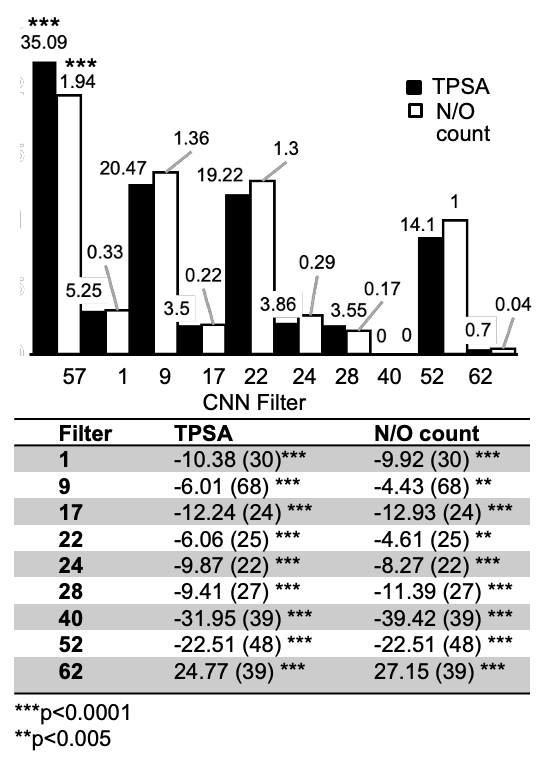


**Supplementary Fig. S5. Means of chemical features in substructures obtained by each filter.** Top: ANOVA comparison between filter 57 and non-inhibitor filters. Bottom: Post-hoc t-test values and degrees of freedom (DF) for each filter in comparison to filter 57. Substructures from filter 57 had higher values than substructures from the other filters in those two measurements. P-values obtained using Bonferroni correction.


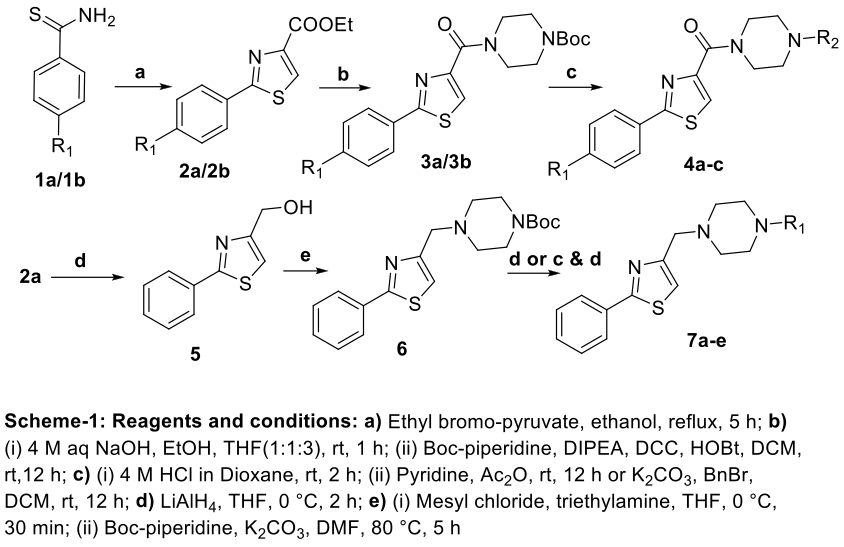


**Supplementary Fig. S6. Synthesis of 2-phenylthiazol-4N-substituted piperazine hybrid molecules.** Reagents and conditions: a) Ethyl bromo-pyruvate, ethanol, reflux, 5h; b) (i) 4M aq NaOH, EtOH, THF(1:1:3), rt, 1h; (ii) Boc-piperidine, DIPEA, DCC, HOBt, DCM, rt, 12h; c) (i) 4M HCl in Dioxane, rt, 2h; (ii) Pyridine, Ac_2_O, rt, 12h or K_2_Co_3_, BnBr, DCM, rt 12h; d) LiAlH_4_, THF, 0 ᵒC, 2h; e) (i) Mesyl chloride, triethylamine, THF, 0 ᵒC, 30 min; (ii) Boc-piperidine, K_2_CO_3_, DMF, 80 ᵒC, 5h.

**Supplementary Figures S7-S16: ^1^H and ^13^C NMR spectra of synthesized compounds.**


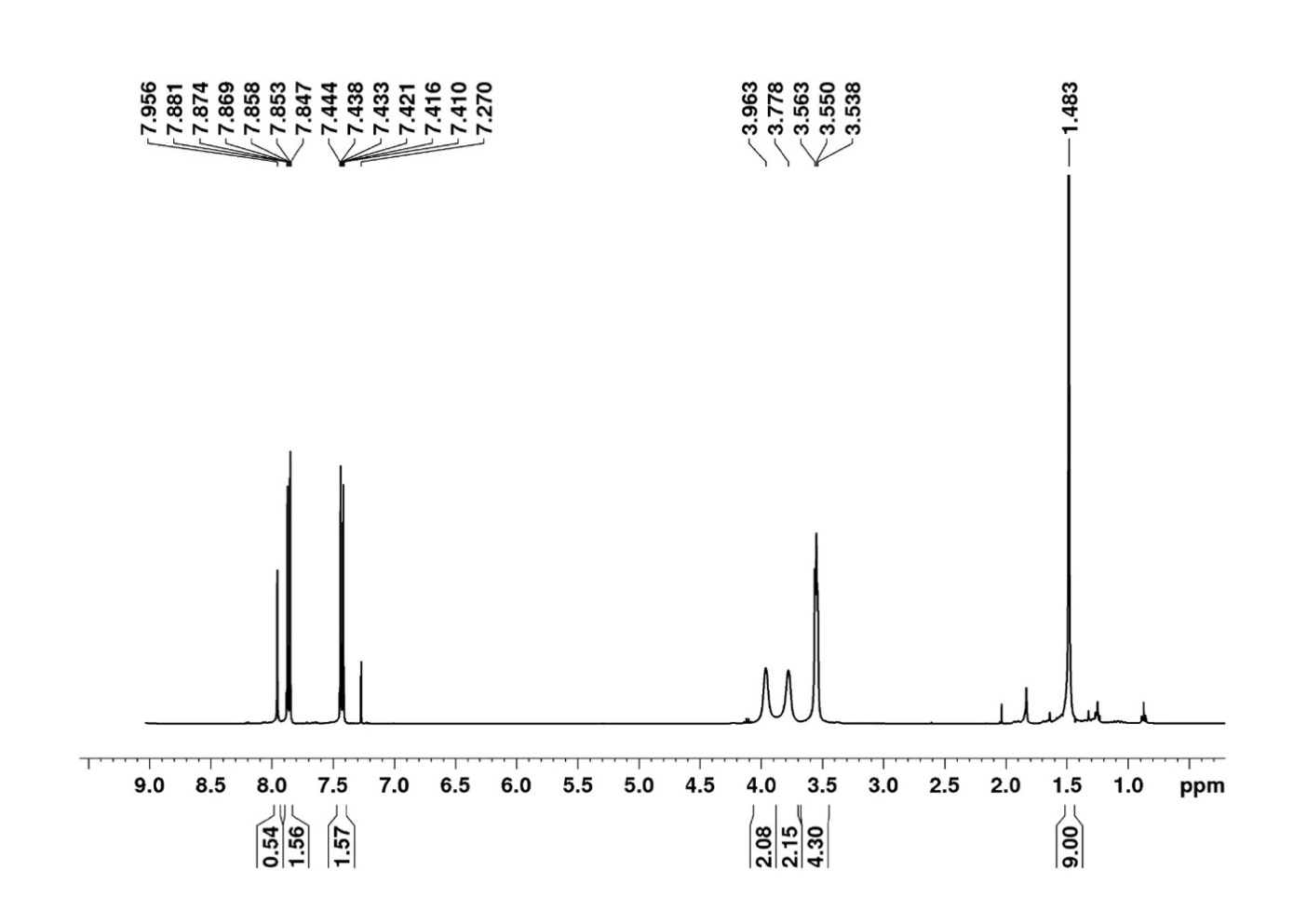

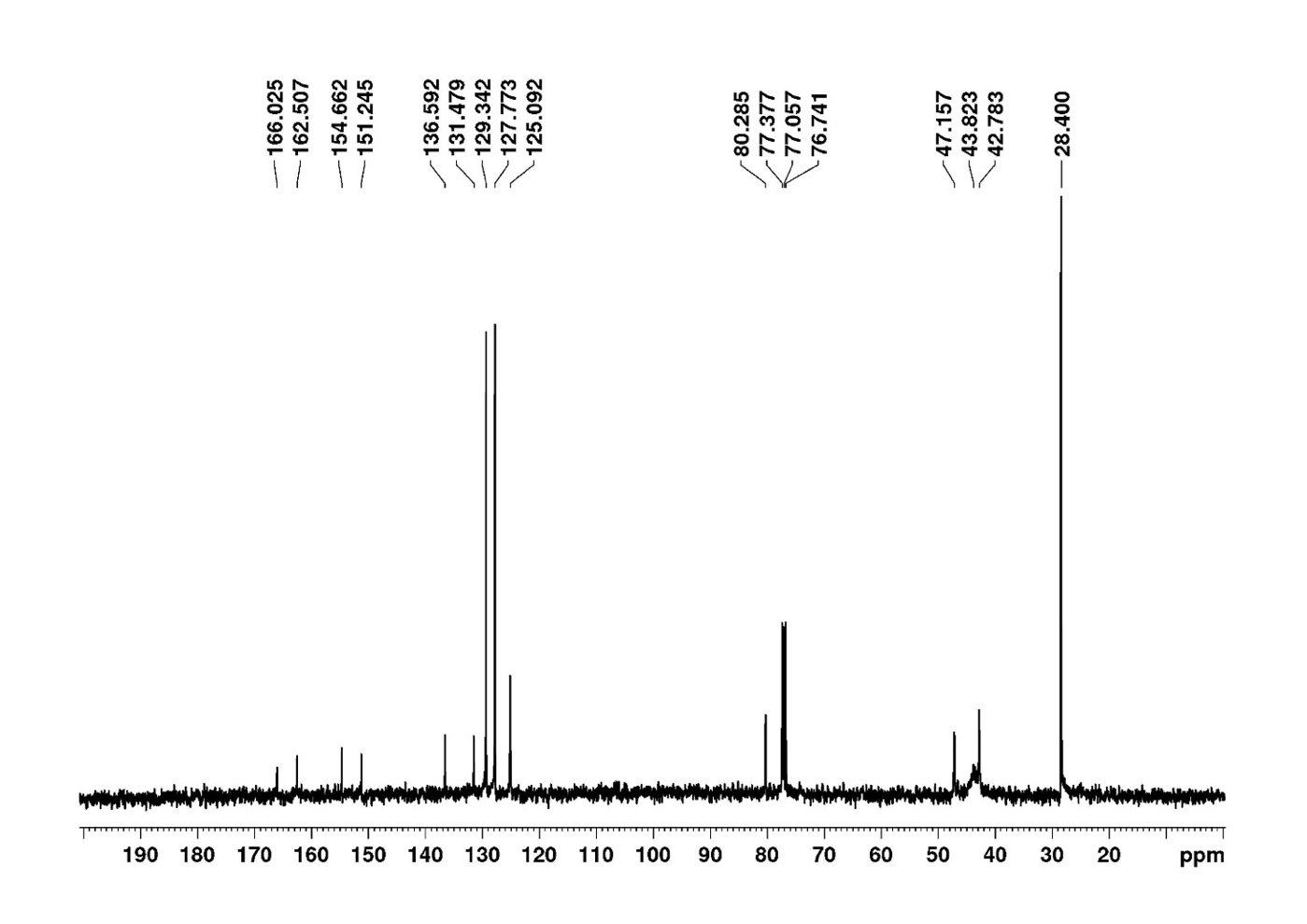


**Supplementary Fig. S7. ^1^H and ^13^C NMR spectra of compound 3b (400MHz, CDCl_3_, 300 K)**


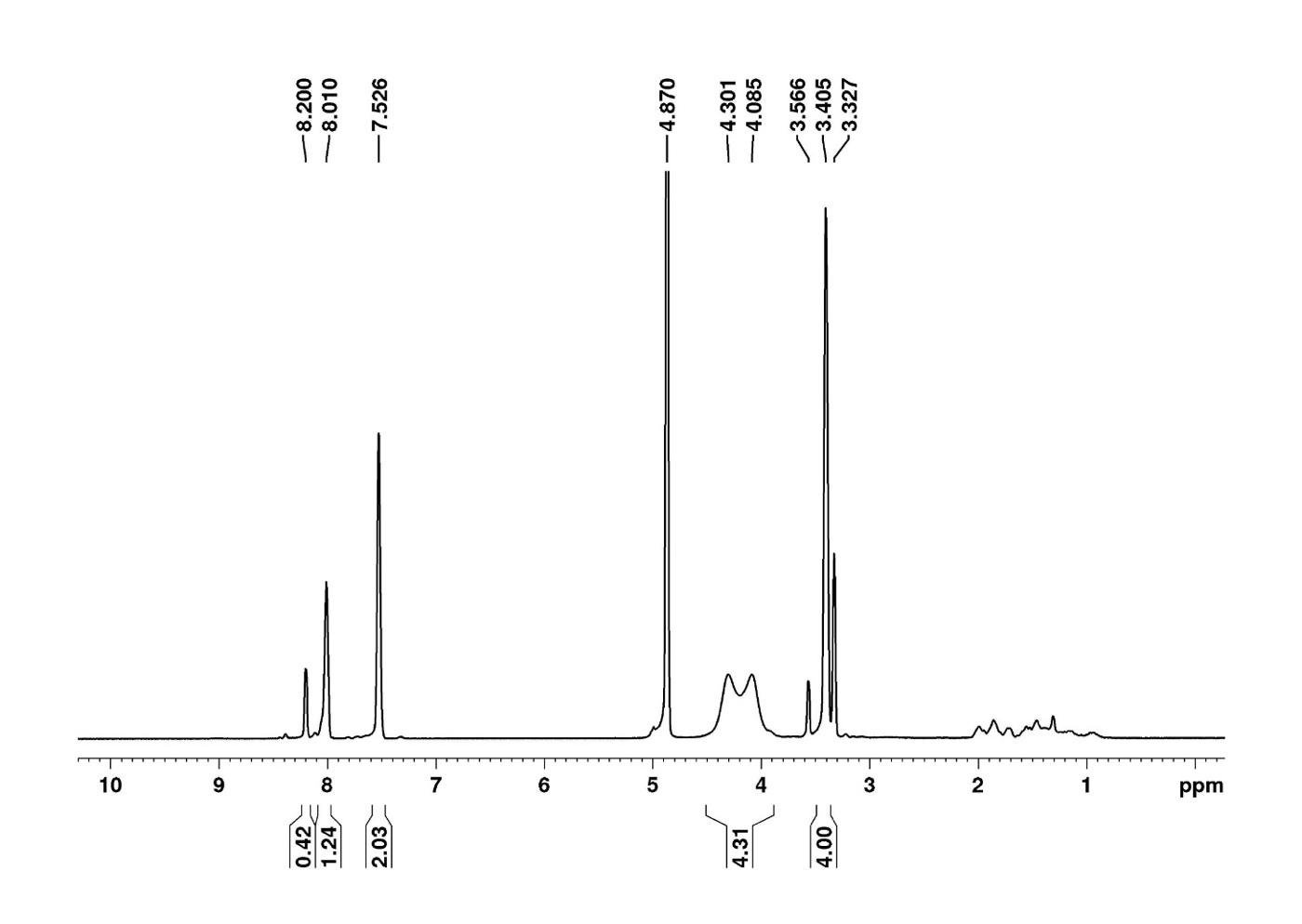

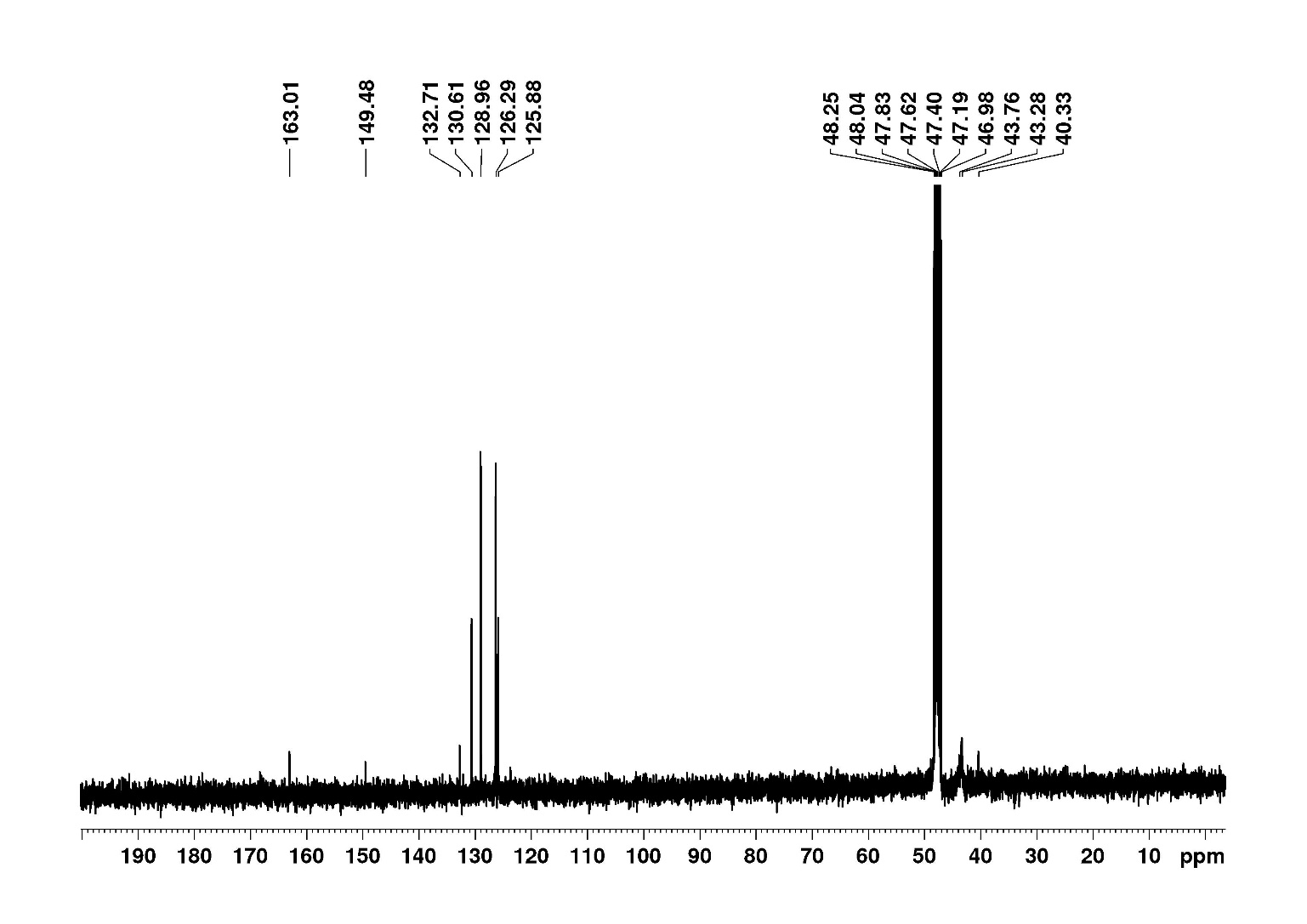


**Supplementary Fig. S8. ^1^H and ^13^C NMR spectra of compound 4a (400MHz, CD_3_OD, 300 K)**


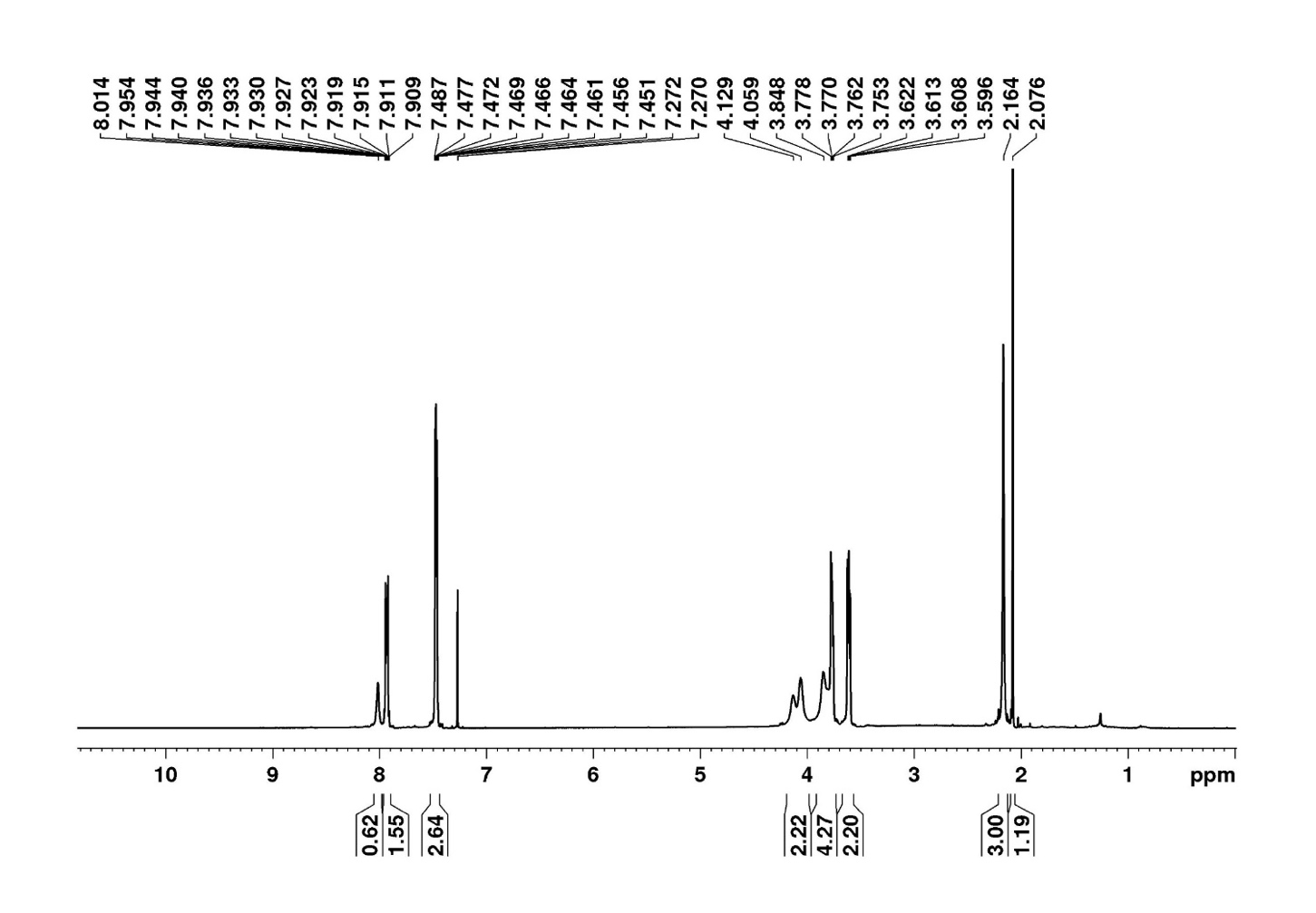

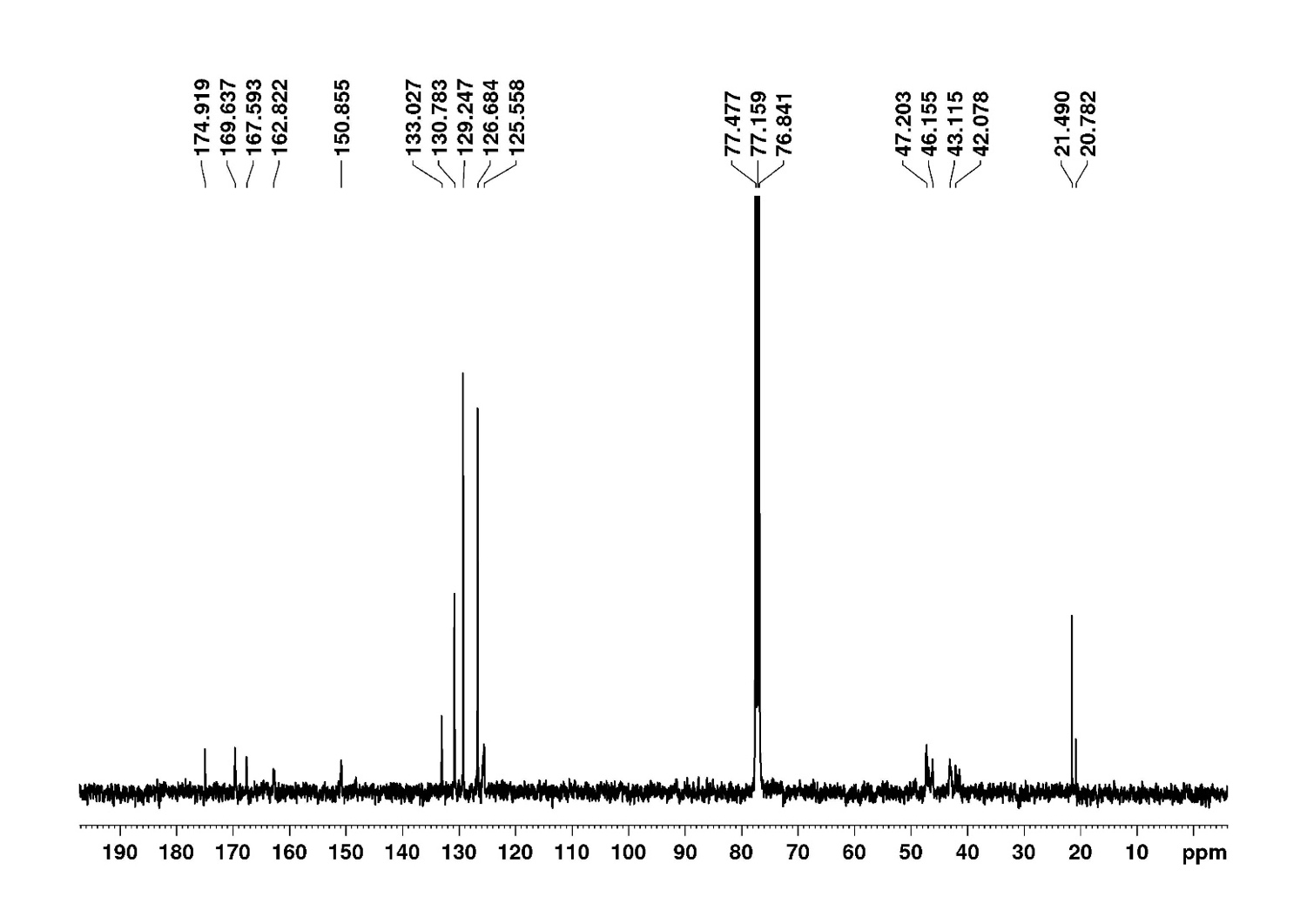


**Supplementary Fig. S9. ^1^H and ^13^C NMR spectra of compound 4b (400MHz, CDCl_3_, 300 K)**


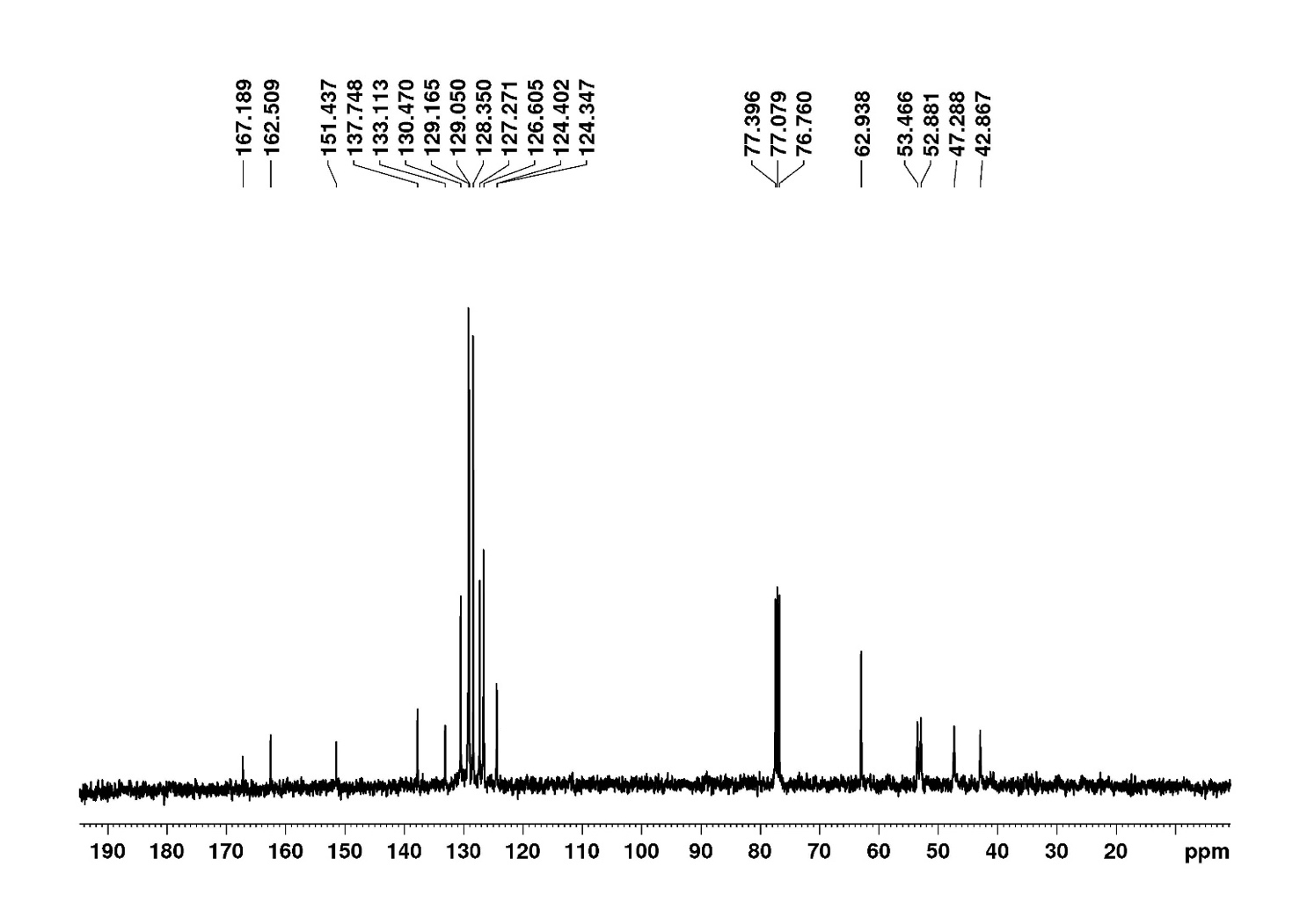


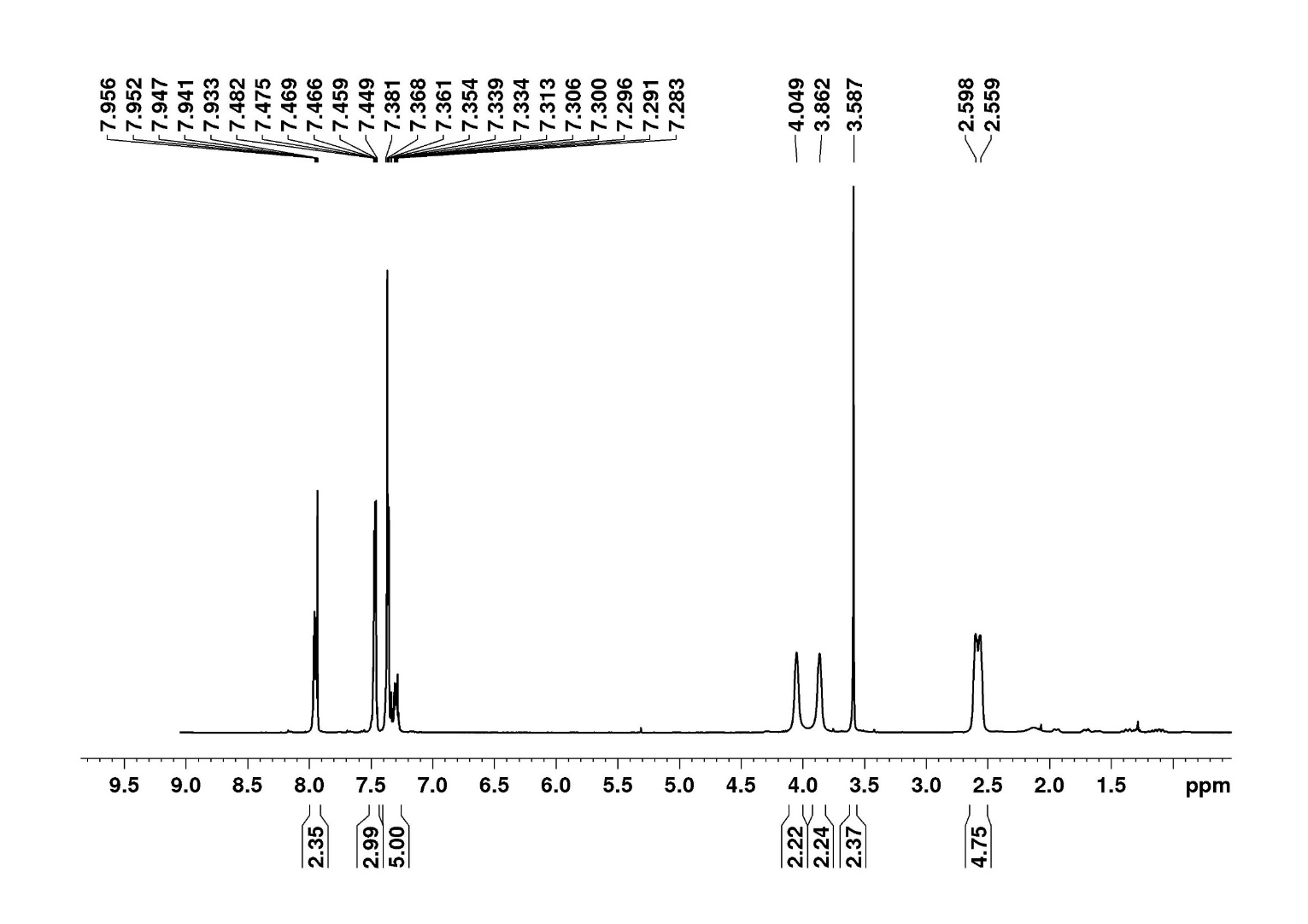


**Supplementary Fig. S10. ^1^H and ^13^C NMR spectra of compound 4c (400MHz, CDCl_3_, 300 K)**


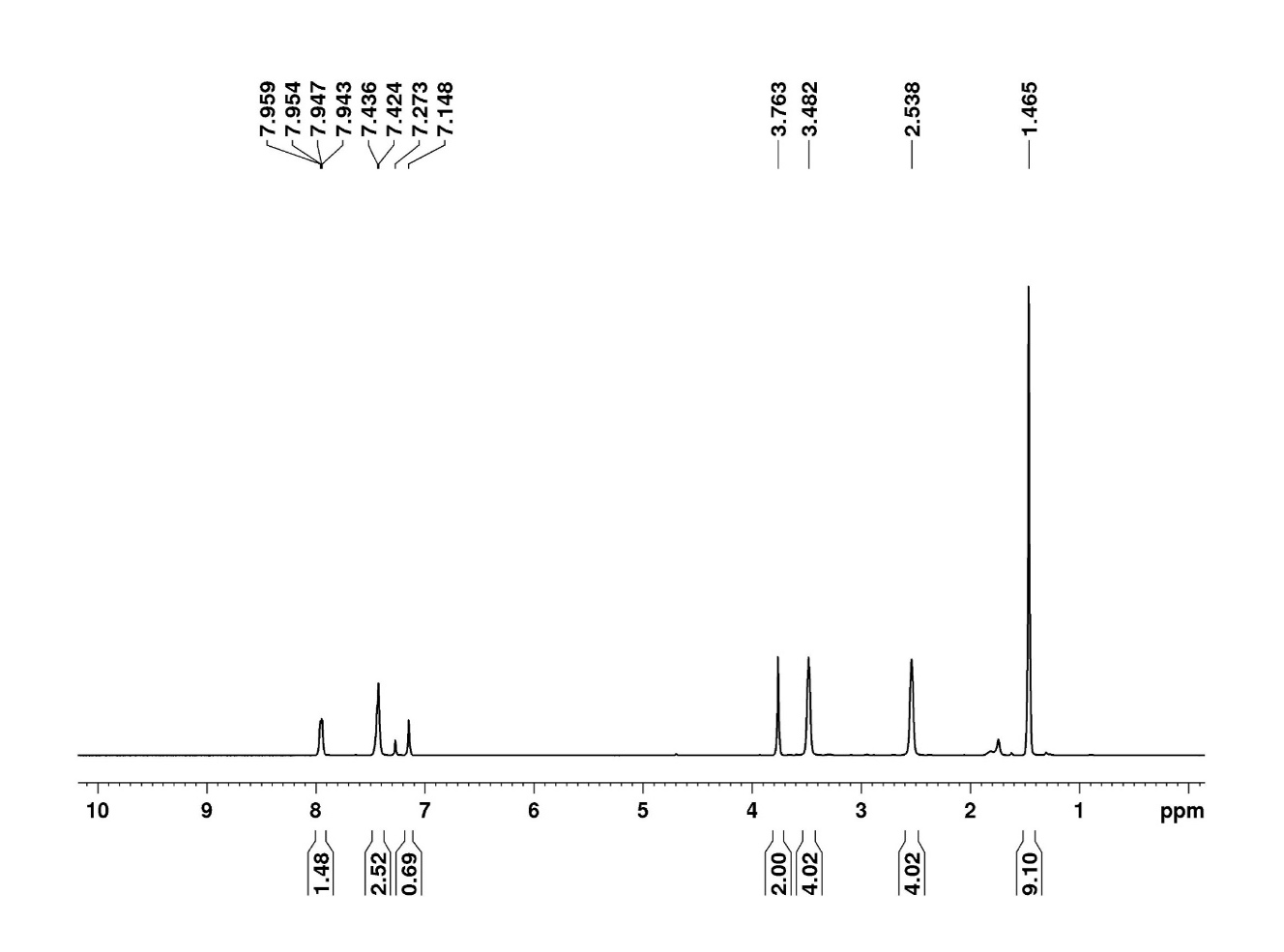

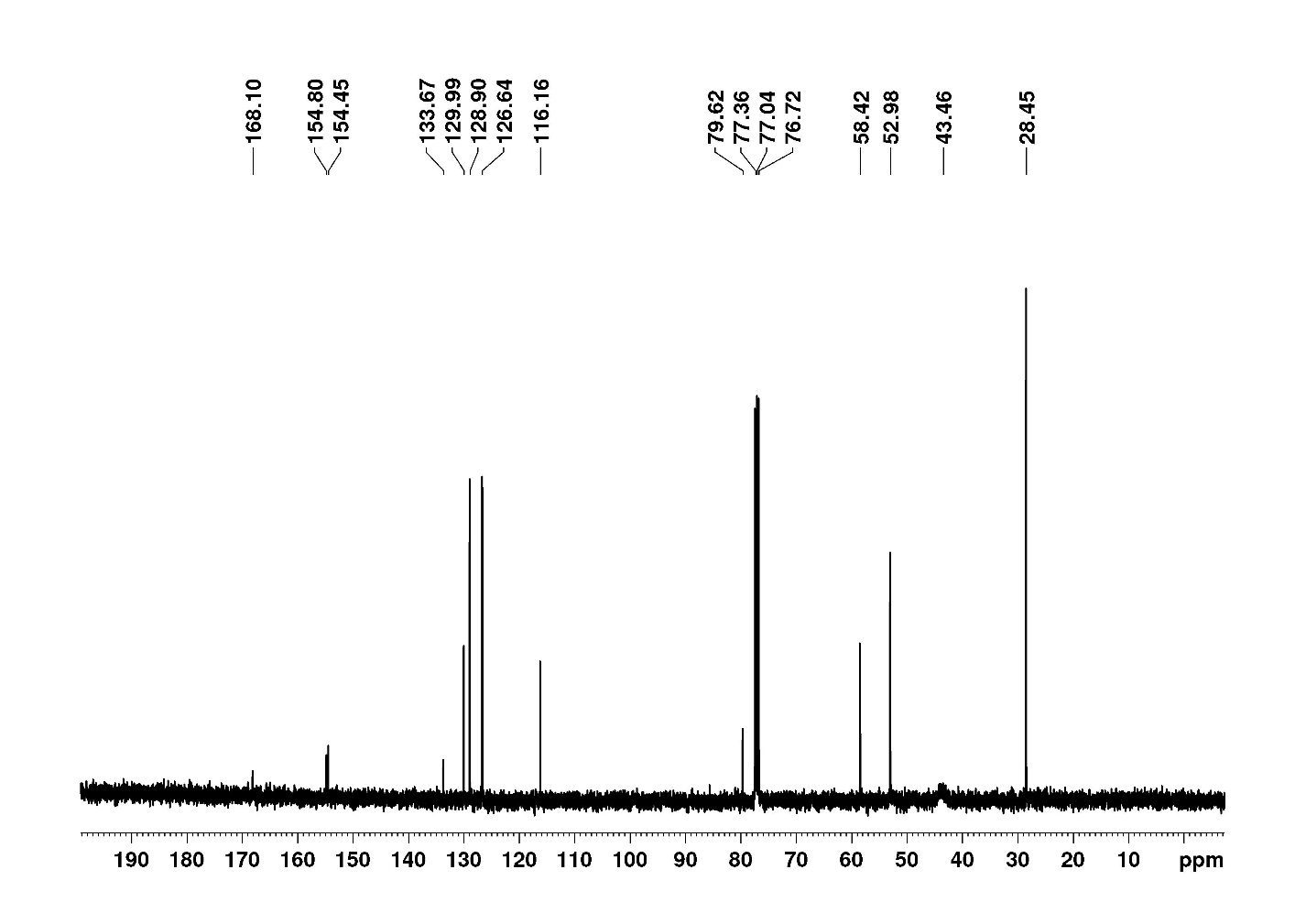


**Supplementary Fig. S11. ^1^H and ^13^C NMR spectra of compound 6 (400MHz, CDCl_3_, 300 K)**


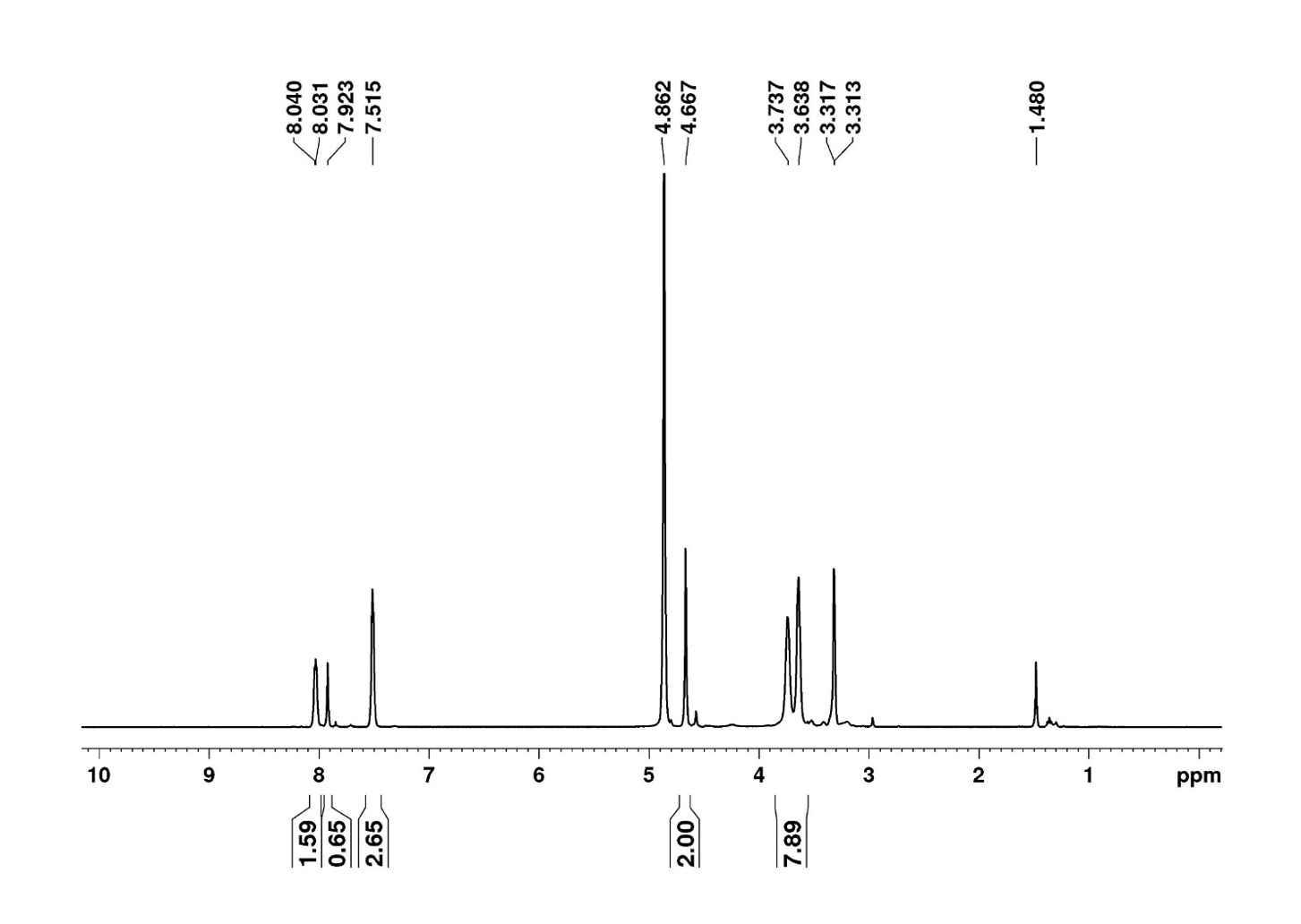

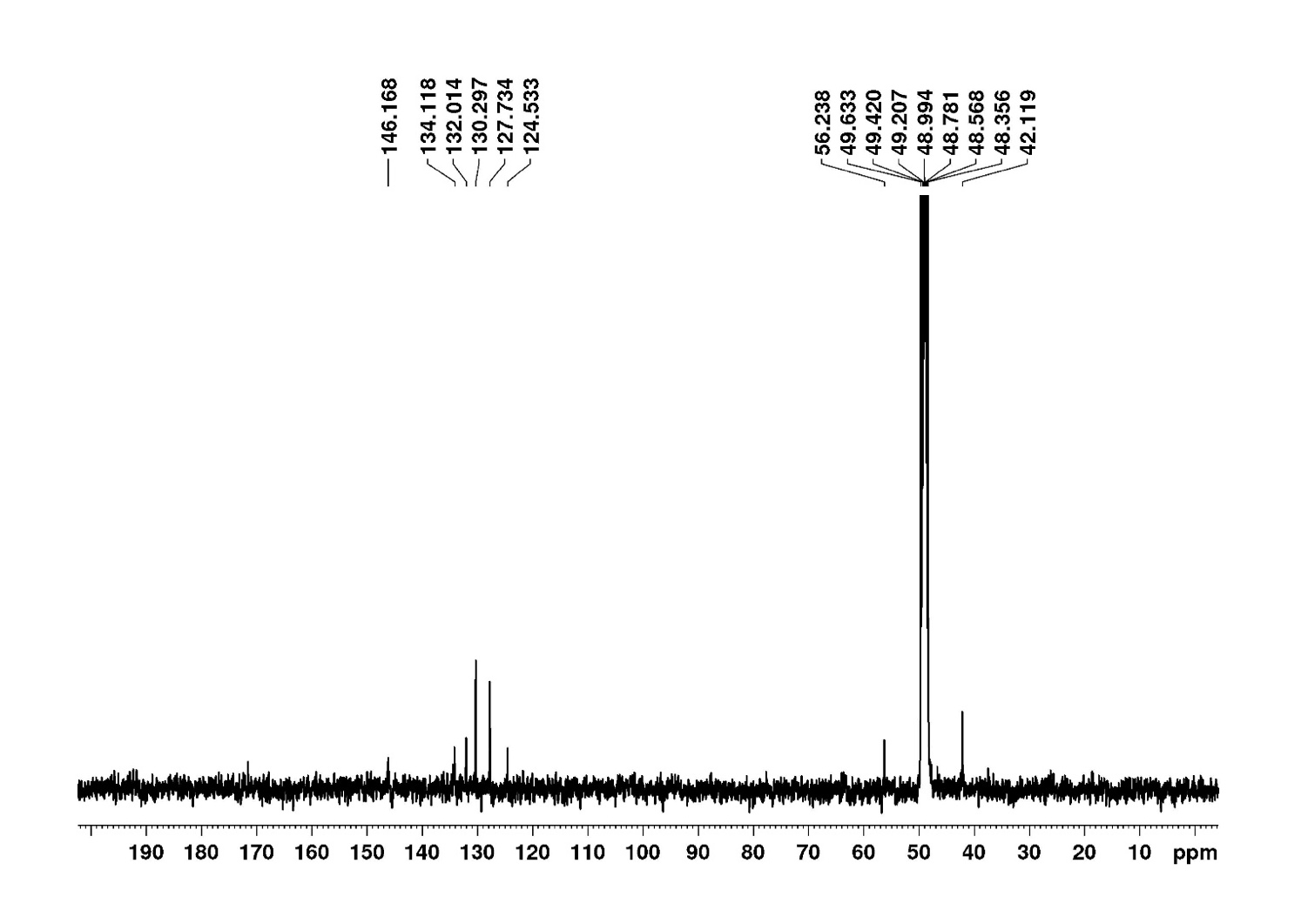


**Supplementary Fig. S12. ^1^H and ^13^C NMR spectra of compound 7a (400MHz, CD_3_OD, 300 K)**


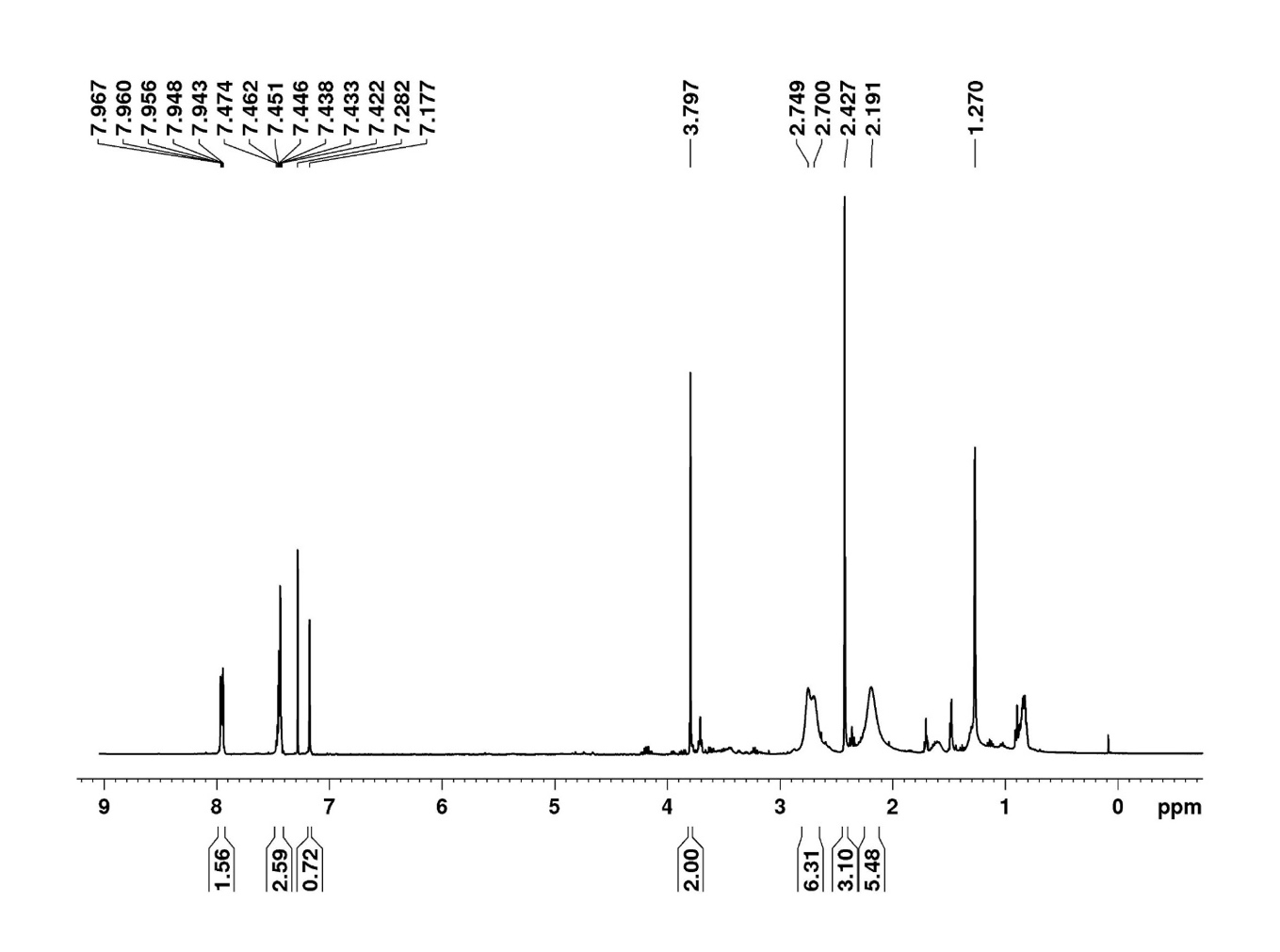

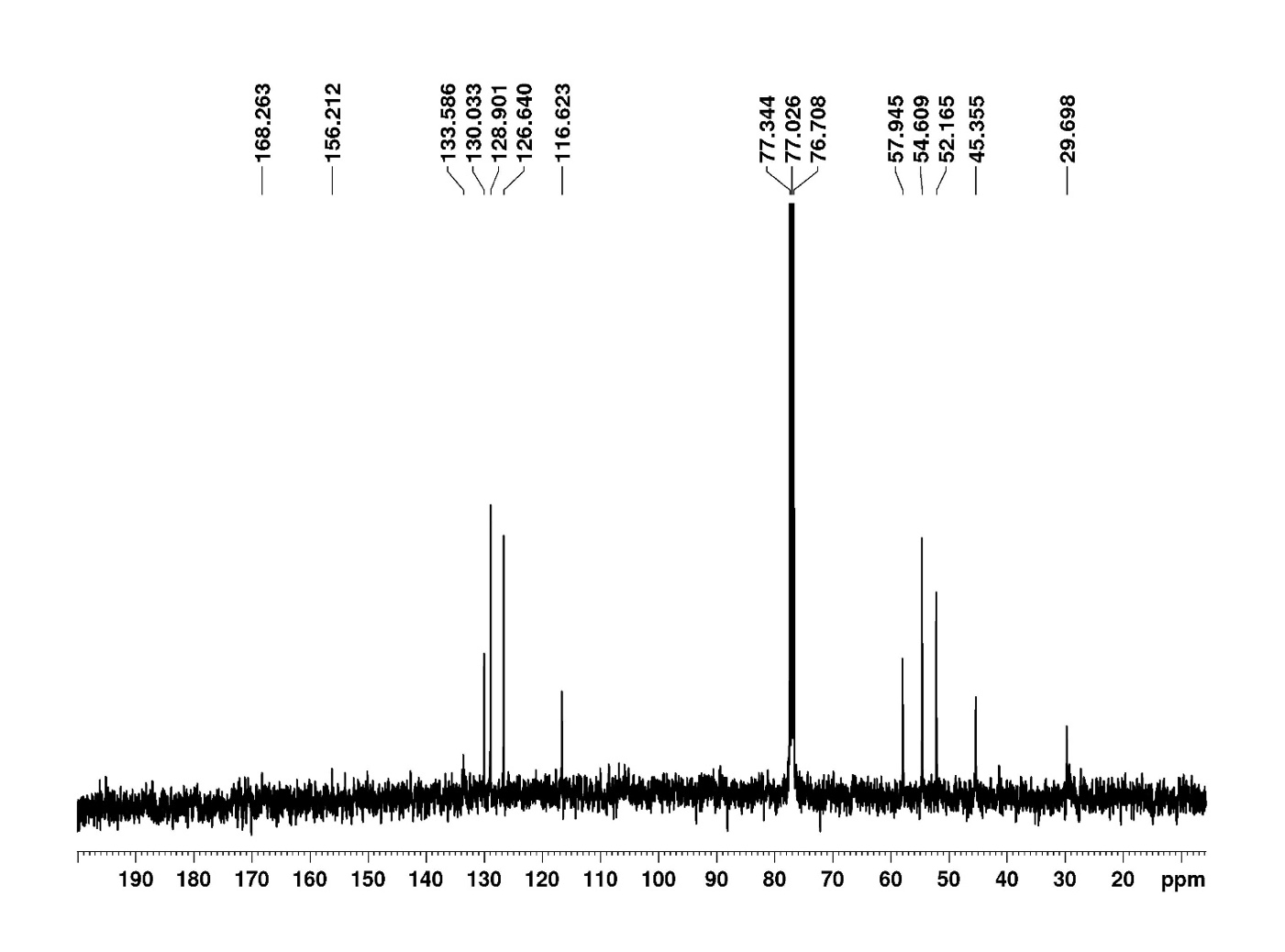


**Supplementary Fig. S13. ^1^H and ^13^C NMR spectra of compound 7b (400MHz, CDCl_3_, 300 K)**


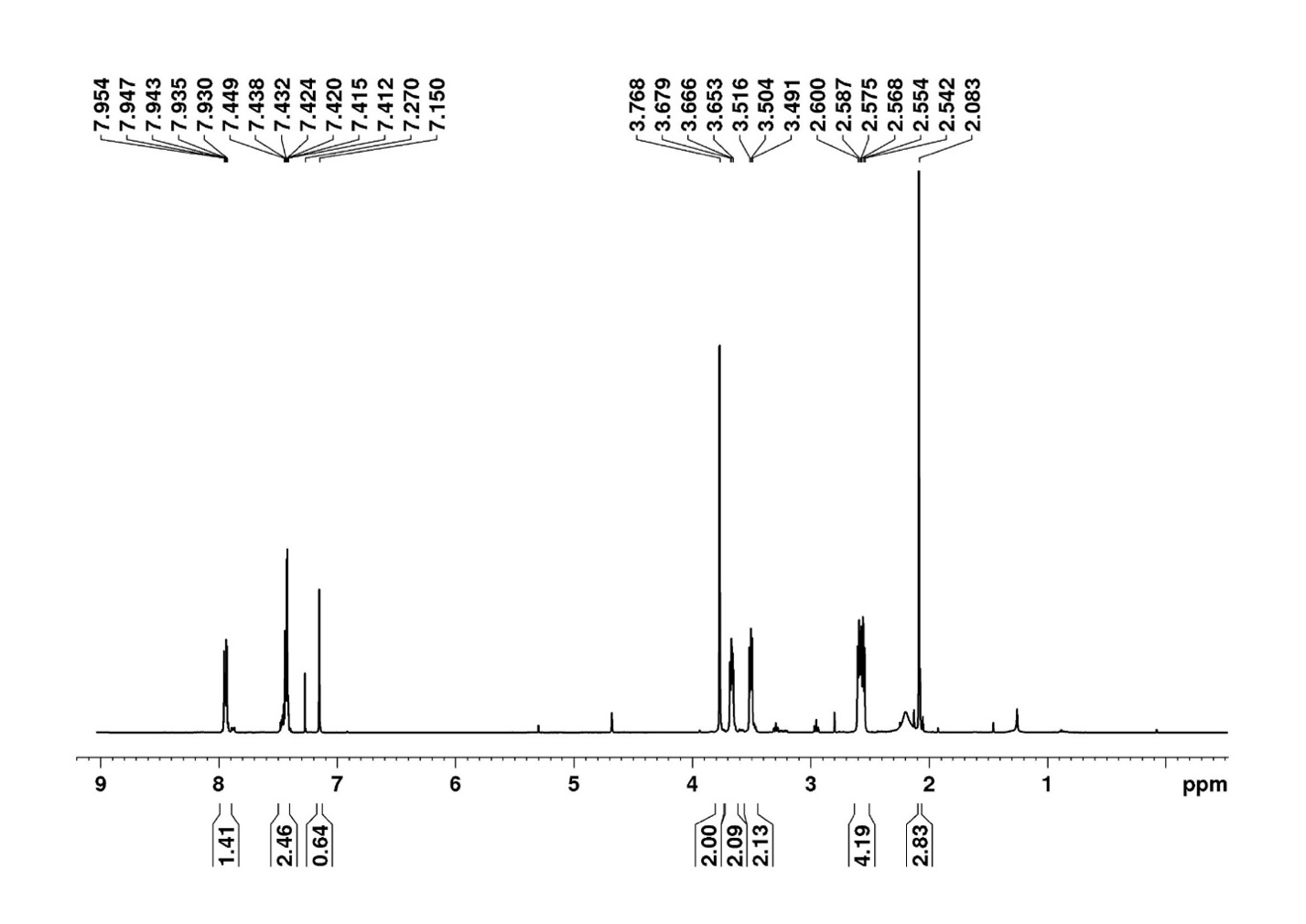

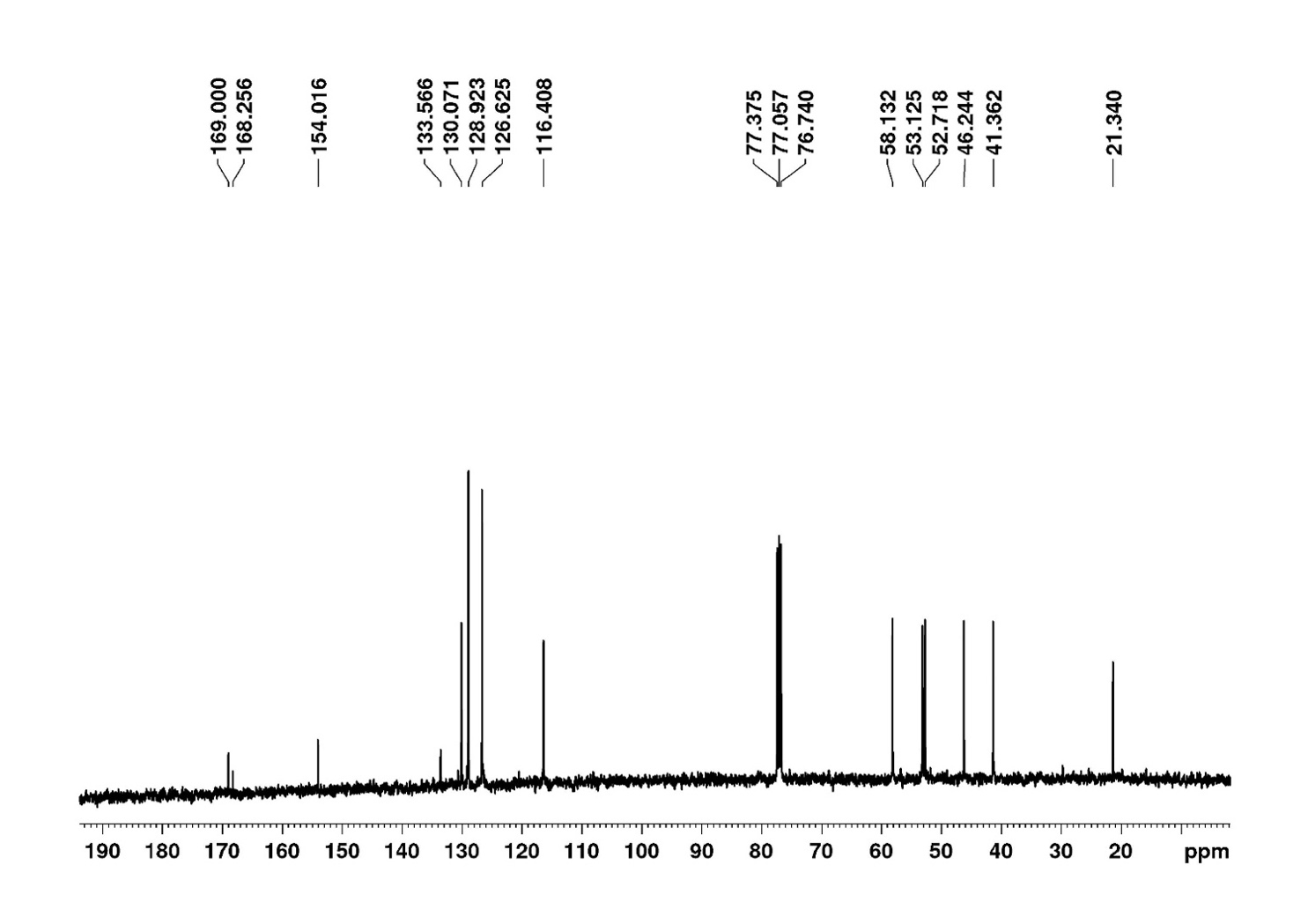


**Supplementary Fig. S14. ^1^H and ^13^C NMR spectra of compound 7c (400MHz, CDCl_3_, 300 K)**


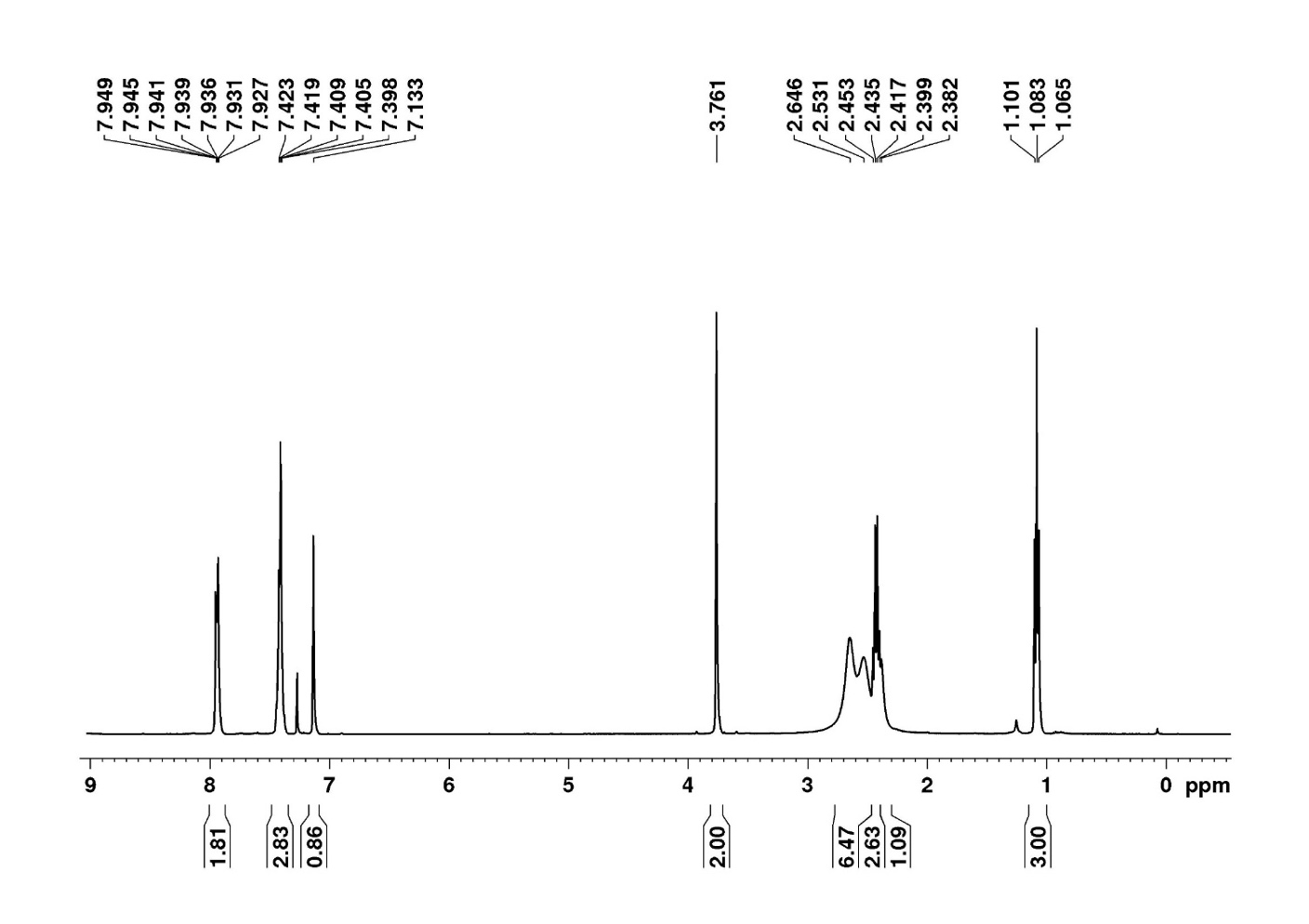

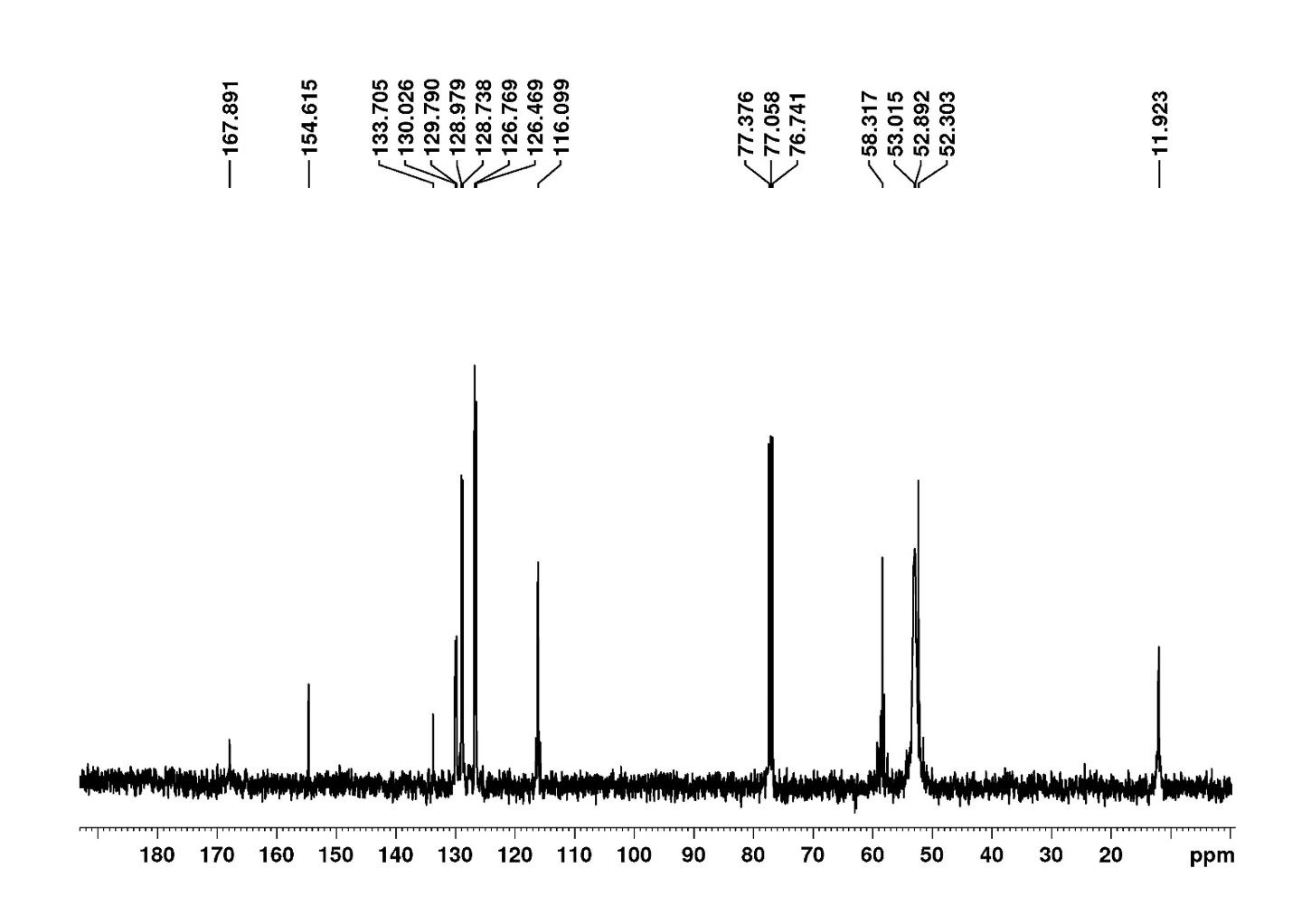


**Supplementary Fig. S15. ^1^H and ^13^C NMR spectra of compound 7d (400MHz, CDCl_3_, 300 K)**


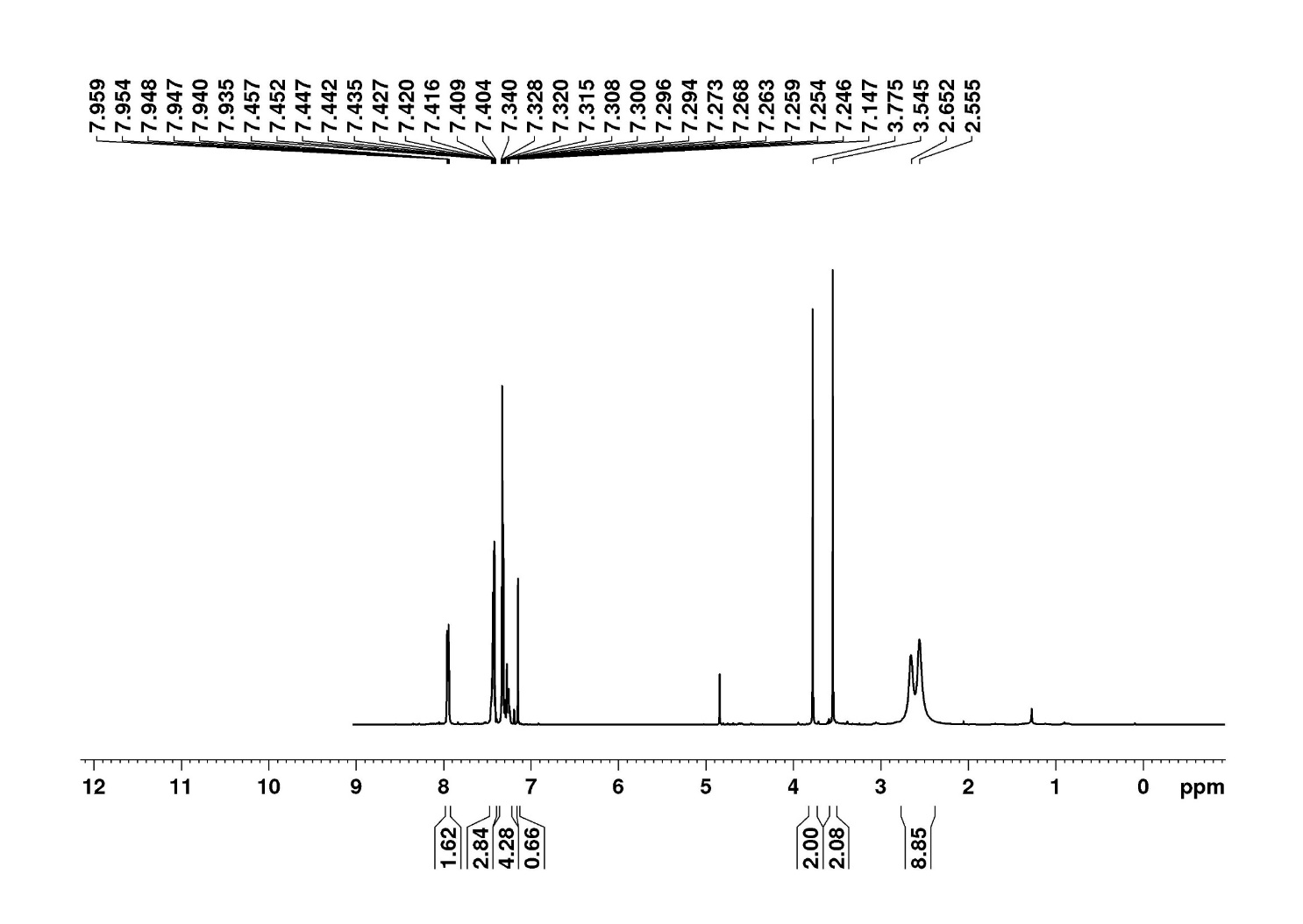

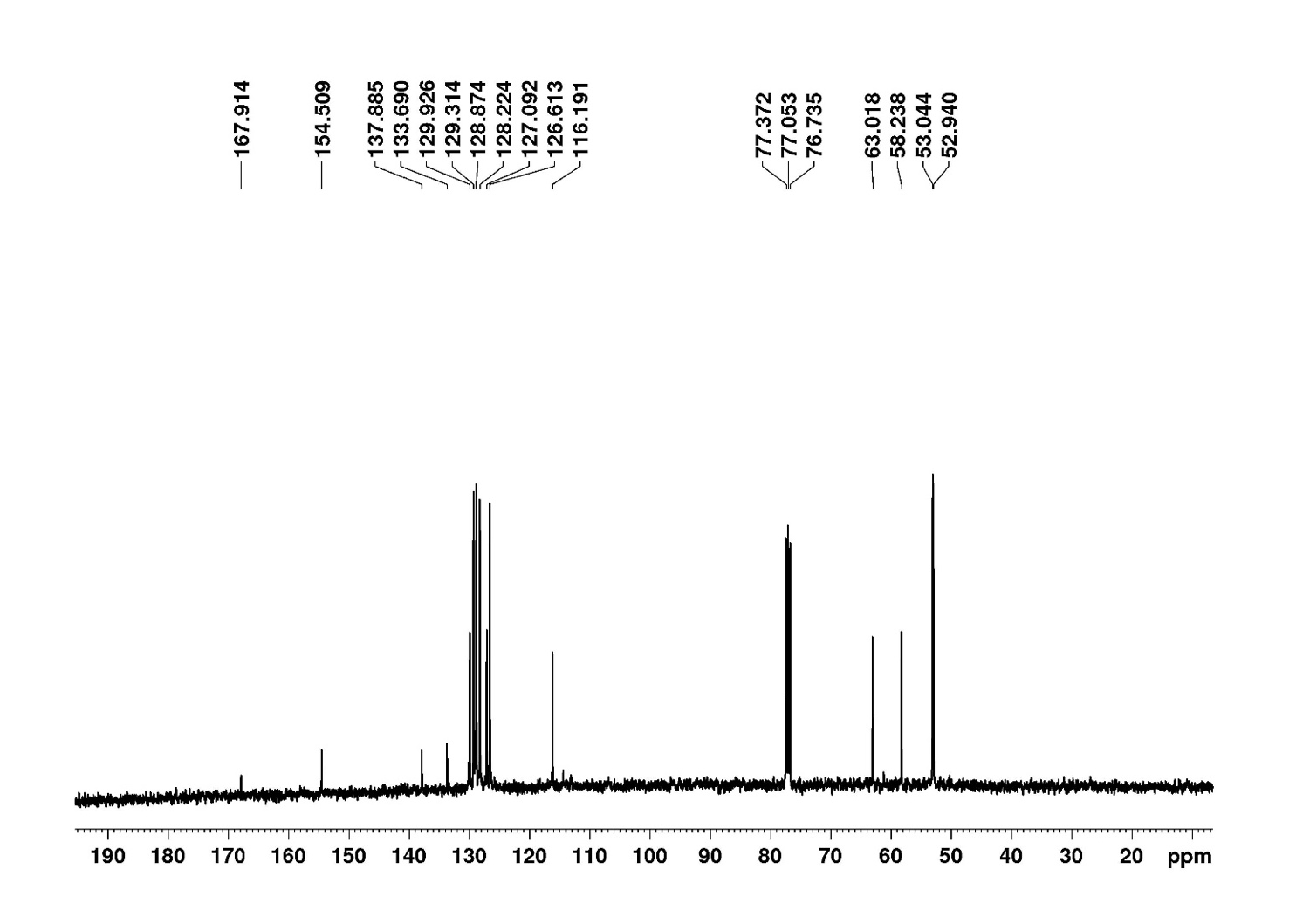


**Supplementary Fig. S16: ^1^H and ^13^C NMR spectra of compound 7e (400MHz, CDCl_3_, 300 K)**

**Characterization Data:**

**1. Ethyl 2-phenylthiazole-4-carboxylate (2a)** R*_f_* 0.42 (Hexane/EtOAc (8:2));^1^H NMR (400 MHz, CDCl_3_) δ 8.17 (s, 1H), 8.01-8.04 (m, 2H), 7.46-7.49 (m, 3H), 4.47 (q, *J*=7.14 Hz, 2H), 1.45 (t, *J*=7.12 Hz, 3H); ^13^C NMR (100 MHz, CDCl_3_) δ 168.5, 160.9, 147.6, 132.3, 130.3, 128.5, 126.6, 126.5, 61.1, 13.9; ESI MS calcd. m/z 233, obs. (M+H) 234.

**2. Ethyl 2-(4-chlorophenyl)thiazole-4-carboxylate (2b)** R*_f_* 0.46 (Hexane/EtOAc (8:2)); ^1^H NMR (500 MHz, CDCl_3_) δ 8.16 (s, 1H), 7.95 (d, *J*=8.48 Hz, 2H), 7.43 (d, *J*=8.58 Hz, 2H), 4.45 (q, *J*=7.14 Hz, 2H), 1.43 (t, *J*=7.13 Hz, 3H); ^13^C NMR (125 MHz, CDCl_3_) δ 168.5, 162.3, 149.2, 137.8, 132.3, 130.2, 129.2, 128.2, 62.6, 15.4; ESI MS calcd. m/z 267, obs. (M+H) 268 & (M+H+2) 270.

**3. tert-butyl 4-(2-phenylthiazole-4-carbonyl)piperazine-1-carboxylate (3a)** R*_f_* 0.32 (Hexane/EtOAc (7:3)); ^1^H NMR (400 MHz, CDCl_3_) δ 7.96 (s, 1H), 7.93-7.95 (m, 2H), 7.45-7.48 (m, 3H), 3.99 (bs, 2H), 3.78 (bs, 2H), 3.45-3.57 (m, 4H), 1.48 (s, 9H); ^13^C NMR (100 MHz, CDCl_3_) δ 167.3, 162.7, 154.7, 151.1, 133.0, 130.6, 129.1, 126.6, 124.9, 80.3, 47.2, 42.8, 33.9, 28.4; ESI MS calcd. m/z 373, obs. (M+H-Boc) 274, (M+Na) 396.

**4. tert-butyl 4-(2-(4-chlorophenyl)thiazole-4-carbonyl)piperazine-1-carboxylate (3b)** R*_f_* 0.36 (hexane/EtOAc (7:3)); ^1^H NMR (400 MHz, CDCl_3_) δ 7.96 (s, 1H), 7.85-7.88 (m, 2H), 7.41-7.44 (m, 2H), 3.96 (bs, 2H), 3.78 (bs, 2H), 3.53-3.56 (m, 4H), 1.48 (s, 9H); ^13^C NMR (100 MHz, CDCl_3_) δ 166.0, 162.5, 154.7, 151.2, 136.6, 131.5, 129.3, 127.8, 125.1, 80.3, 47.2, 43.8, 42.7, 28.4; ESI MS calcd. m/z 407, obs. (M+H-Boc) 308, (M+Na) 430, (M+K) 446.

**5.** **(2-phenylthiazol-4-yl)(piperazin-1-yl)methanone (4a)** R*_f_* 0.32 (chloroform/methanol (9:1)); ^1^H NMR (400 MHz, CD_3_OD) δ 8.20 (s, 1H), 8.01 (bs, 2H), 7.53 (bs, 3H), 4.30 (bs, 2H), 4.08 (bs, 2H), 3.40-3.57 (m, 4H); ^13^C NMR (100 MHz, CD_3_OD) δ 168.2,163.0, 149.5, 132.7, 130.6, 128.9, 126.3, 125.9, 43.8, 43.3, 40.3; ESI MS calcd. m/z 273, obs. (M+H) 274.

**6. 1-(4-(2-phenylthiazole-4-carbonyl)piperazin-1-yl)ethan-1-one (4b)** R*_f_* 0.52 (chloroform/methanol (9:1)); ^1^H NMR (400 MHz, CDCl_3_) δ 8.01 (s, 1H), 7.91-7.95 (m, 2H), 7.45-7.49 (m, 3H), 4.06-4.13 (m, 2H), 3.75-3.85 (m, 4H), 3.60-3.62 (m, 2H), 2.16 (s, 3H); ^13^C NMR (100 MHz, CDCl_3_) δ 174.9, 169.6, 162.8, 150.8, 133.0, 130.8, 129.2, 125.6, 47.2, 46.2, 43.1, 42.2, 21.5; ESI MS calcd. m/z 315, obs. (M+H) 316, (M+Na) 338.

**7. (4-benzylpiperazin-1-yl)(2-phenylthiazol-4-yl)methanone (4c)** R*_f_* 0.56 (chloroform/methanol (9:1)); ^1^H NMR (400 MHz, CDCl_3_) δ 7.94-7.96 (m, 2H), 7.93 (s, 1H), 7.44-7.48 (m, 3H), 7.28-7.38 (m, 5H), 4.05 (bs, 2H), 3.86 (bs, 2H), 3.59 (s, 2H), 2.56-2.60 (m, 4H); ^13^C NMR (100 MHz, CDCl_3_) δ 167.2, 162.5, 151.4, 137.7, 133.1, 130.5, 129.2, 129.1, 128.4, 127.3, 126.6, 124.4, 124.3, 62.9, 53.5, 52.9, 47.3, 42.9; ESI MS calcd. m/z 363, obs. (M+H) 364.

**8. (2-phenylthiazol-4-yl)methanol (5)** R*_f_* 0.21 (Hexane/EtOAc (7:3)); ^1^H NMR (400 MHz, CDCl_3_) δ 8.03-8.05 (m, 2H), 7.52-7.56 (m, 3H), 7.29 (t, *J*=0.82 Hz, 1H), 4.94 (d, *J*=0.86 Hz 2H), 3.25 (bs, 1H); ^13^C NMR (100 MHz, CDCl_3_) δ 168.9, 157.3, 133.4, 130.2, 128.9, 126.6, 114.7, 60.9; ESI MS calcd. m/z 191, obs. (M+H) 192.

**9. tert-butyl 4-((2-phenylthiazol-4-yl)methyl)piperazine-1-carboxylate (6)** R*_f_* 0.31 (Hexane/EtOAc (7:3)); ^1^H NMR (400 MHz, CDCl_3_) δ 7.94-7.95 (m, 2H), 7.42-7.44 (m, 3H), 7.15(s, 1H), 3.76 (s, 2H), 3.48 (bs, 4H), 2.54 (bs, 4H),1.47 (s, 9H); ^13^C NMR (100 MHz, CDCl_3_) δ 168.1, 154.8, 154.5, 133.7, 129.9, 128.9, 126.6, 116.2, 79.6, 58.4, 52.9, 43.5, 28.5. ESI MS calcd. m/z 359, obs. (M+H) 360.

**10.** **2-phenyl-4-(piperazin-1-ylmethyl)thiazole (7a)** R*_f_* 0.29 (chloroform/methanol (9:1)); ^1^H NMR (400 MHz, CD_3_OD) δ 8.03-8.04 (m, 2H), 7.92 (s, 1H), 7.52 (bs, 3H), 4.67 (s, 2H), 3.64-3.74 (m, 8H); ^13^C NMR (100 MHz, CD_3_OD) δ 146.2, 134.1, 132.0, 130.3, 127.7, 124.5, 56.2, 42.1; ESI MS calcd. m/z 259, obs. (M+H) 260.

**11. 4-((4-methylpiperazin-1-yl)methyl)-2-phenylthiazole (7b)** R*_f_* 0.32 (chloroform/methanol (9:1)) ^1^H NMR (400 MHz, CDCl_3_) δ 7.94-7.97 (m, 2H), 7.42-7.47 (m, 3H), 7.18 (s, 1H), 3.79 (s, 2H), 2.70-2.75 (m, 4H), 2.48 (s, 2H), 2.19 (bs, 4H); ^13^C NMR (100 MHz, CDCl_3_) δ 168.3, 156.2, 133.6, 130.3, 128.9, 126.6, 116.6, 57.9, 54.6, 52.2, 45.3, 29.7; ESI MS calcd. m/z 273, obs. (M+H) 274.

**12. 1-(4-((2-phenylthiazol-4-yl)methyl)piperazin-1-yl)ethan-1-one (7c)** R*_f_* 0.40 (chloroform/methanol (9:1)) ^1^H NMR (400 MHz, CDCl_3_) δ 7.93-7.95 (m, 2H), 7.41-7.45 (m, 3H), 7.15 (s, 1H), 3.77 (s, 2H), 3.65-3.68 (m, 2H), 3.49-3.52 (m, 2H), 2.54-2.60 (m, 4H), 2.08 (s, 3H); ^13^C NMR (100 MHz, CDCl_3_) δ 169.0, 168.3, 154.0, 133.6, 130.0, 128.9, 126.6, 116.4, 58.1, 53.1, 52.7, 46.2, 41.4, 21.3; ESI MS calcd. m/z 301, obs. (M+H) 302, (M+Na) 324.

**13. 4-((4-ethylpiperazin-1-yl)methyl)-2-phenylthiazole (7d)** R*_f_* 0.36 (chloroform/methanol (9:1)); ^1^H NMR (400 MHz, CDCl_3_) δ 7.93-7.95 (m, 2H), 7.40-7.42 (m, 3H), 7.13 (s, 1H), 3.76 (s, 2H), 2.38-2.65 (m, 10H), 1.08 (t, *J*=7.2 Hz, 3H); ^13^C NMR (100 MHz, CDCl_3_) δ 167.9, 154.6, 133.7, 130.0, 129.8, 128.9, 128.7, 126.8, 126.5, 116.1, 58.3, 53.0, 52.9, 52.3, 11.9; ESI MS calcd. m/z 287, obs. (M+H) 288.

**14. 4-((4-benzylpiperazin-1-yl)methyl)-2-phenylthiazole (7e)** R*_f_* 0.39 (chloroform/methanol (9:1)); ^1^H NMR (400 MHz, CDCl_3_) δ 7.94-7.96 (m, 2H), 7.40-7.46 (m, 3H), 7.25-7.34 (m, 5H), 7.15 (s, 1H), 3.78 (s, 2H), 3.55 (s, 2H), 2.65 (bs, 4H), 2.56 (bs, 4H); ^13^C NMR (100 MHz, CDCl_3_) δ 167.9, 154.5, 137.9, 133.9, 129.9, 129.3, 128.8, 128.2, 127.1, 126.6, 116.2, 63.0, 58.2, 53.0, 52.9; ESI MS calcd. m/z 349, obs. (M+H) 350.

1. Bisong, E., Google Colaboratory. In *Building Machine Learning and Deep Learning Models on Google Cloud Platform.* , Apress, Berkeley, CA: 2019.
